# Machine Learning–Based Prediction of Major Postoperative Complications After Noncardiac Surgery Under General Anesthesia Using Routinely Available Electronic Health Record Data: Retrospective Multicenter Development and Validation Study

**DOI:** 10.2196/91315

**Published:** 2026-10-21

**Authors:** Takuya Nishino, Hiroshi Mase, Chol Kim, Shinji Sugita, Yukihiro Kondo, Masashi Ishikawa

**Affiliations:** 1 Department of Health Policy and Management, Nippon Medical School, Bunkyo-ku, Tokyo, Japan; 2 Department of Anesthesiology and Pain Medicine, Nippon Medical School, 1-1-5 Sendagi, Bunkyo-ku, Tokyo, 113-8603, Japan; 3 Department of Surgical Intensive Care, Nippon Medical School, Bunkyo-ku, Tokyo, Japan; 4 Department of Anesthesiology, Nippon Medical School Chiba Hokusoh Hospital, Chiba, Japan; 5 Department of Anesthesiology, Nippon Medical School Musashi Kosugi Hospital, Kawasaki, Kanagawa, Japan; 6 Department of Urology, Nippon Medical School, Tokyo, Japan

**Keywords:** perioperative risk, prediction model, postoperative complications, anesthesia, machine learning, electronic health records

## Abstract

**Background:**

Postoperative complications after noncardiac surgery under general anesthesia remain a major cause of morbidity, mortality, and resource-intensive care. Conventional risk assessment tools rely mainly on preoperative information and may not capture perioperative physiological and procedural factors associated with postoperative risk.

**Objective:**

This retrospective multicenter observational study aimed to develop and validate electronic health record (EHR)–based machine learning (ML) models for predicting major postoperative complications and to evaluate their discrimination, overall probabilistic performance, calibration, and potential clinical utility.

**Methods:**

We analyzed 35,702 adults who underwent noncardiac surgery under general anesthesia at 3 tertiary hospitals between January 2021 and March 2025. Preoperative, intraoperative, and early postoperative variables were extracted from diagnosis procedure combination (DPC) claims, EHRs, and anesthesia information systems. The primary outcome was a 30-day composite of in-hospital death, unplanned reoperation, prolonged mechanical ventilation, new initiation of renal replacement therapy, and postoperative infection. Logistic regression (LR), extreme gradient boosting (XGB), and light gradient boosting machine (LGBM) models were developed using preoperative variables alone or with 7 additional intraoperative and early postoperative variables. Leave-one-facility-out internal-external cross-validation (IECV) was the primary validation framework, with a stratified random 80:20 hold-out analysis as secondary validation. Performance was assessed using the area under the receiver operating characteristic curve (AUROC), area under the precision-recall curve (PR-AUC), Brier score, scaled Brier score, calibration measures, and decision curve analysis.

**Results:**

The primary composite outcome occurred in 3884 of 35,702 patients (10.9%). In pooled IECV, perioperative models consistently outperformed the corresponding preoperative models. AUROC increased from 0.784‐0.791 to 0.810‐0.817, PR-AUC increased from 0.356‐0.361 to 0.405‐0.413, Brier scores decreased from 0.082‐0.083 to 0.078‐0.080, and scaled Brier scores increased from 0.144‐0.151 to 0.175‐0.191. Calibration varied across facilities; pooled calibration slopes for the perioperative models ranged from 0.875 to 0.902, and calibration intercepts ranged from −0.367 to −0.222, indicating overly extreme predictions and overall risk overestimation. Exploratory decision curve analysis generally showed greater net benefit for perioperative models across threshold probabilities of 0.01‐0.30, although the curves overlapped substantially. Secondary and sensitivity analyses were consistent with the primary findings.

**Conclusions:**

Adding routinely available intraoperative and early postoperative variables produced modest but consistent improvements in postoperative risk prediction compared with preoperative variables alone. However, facility-specific variation in calibration limits direct transportability of absolute risk estimates. The models may support prioritization for postoperative clinical reassessment, but exploratory decision curve analysis did not establish a specific intervention or triage threshold. Local recalibration and prospective workflow evaluation are required before clinical implementation.

## Introduction

Approximately 250 million surgical procedures are performed worldwide each year, and this number continues to increase with population aging and expanding surgical indications [1]. As surgical volume rises, the absolute burden of postoperative complications is also expected to grow [2,3]. Postoperative mortality, often attributable to complications such as acute kidney injury, respiratory failure, and major cardiac events, remains a major global health concern, with more than 4 million deaths occurring within 30 days after surgery annually [4-7]. Importantly, multiple studies have demonstrated that early identification of patients at elevated postoperative risk can inform intraoperative and postoperative management strategies, thereby preventing complications or enabling earlier intervention [8-11]. Accordingly, accurate perioperative risk stratification is essential for timely and effective clinical decision-making. To support perioperative risk assessment, several established approaches are commonly used. The American Society of Anesthesiologists Physical Status classification (ASA-PS) provides a global assessment of preoperative physical status, whereas the Revised Cardiac Risk Index estimates perioperative cardiac risk, and the American College of Surgeons Surgical Risk Calculator provides individualized estimates of postoperative outcomes [12-14]. These approaches primarily rely on preoperative patient characteristics, comorbidities, and procedural information. Consequently, they may have limited ability to capture dynamic physiological changes occurring during surgery and the early postoperative period.

In recent years, machine learning (ML) approaches have increasingly been applied to clinical data to improve risk prediction across a wide range of medical domains. Unlike traditional regression-based models, ML algorithms can incorporate large numbers of heterogeneous variables and flexibly model nonlinear relationships and higher-order interactions among predictors [15]. These characteristics enable automated risk scoring systems that leverage high-dimensional clinical data and may outperform conventional approaches in complex clinical settings. In the perioperative context, ML-based decision support tools also have the potential to assist anesthesiologists and surgical teams—particularly less experienced clinicians—by providing objective, data-driven risk estimates to complement clinical judgment [16]. Previous studies have developed ML models to predict postoperative outcomes using demographic characteristics, comorbidities, and laboratory data available preoperatively in single-center electronic health record (EHR) cohorts [17,18], while a large multicenter model based on preoperative EHR data has also been reported [19]. Another study incorporated detailed intraoperative physiological time-series data [20]. Although these studies demonstrate the potential of ML for perioperative risk prediction, the transportability of these models across hospitals with differing patient populations, surgical case mix, and clinical practices remains an important consideration. Integrating routinely available perioperative information may provide additional predictive value while facilitating reproducible implementation across institutions.

In contrast, this study aimed to develop and validate pragmatic multicenter ML-based prediction models using routinely available perioperative data extracted from EHRs, administrative claims, and anesthesia information systems. By focusing on variables consistently recorded across hospitals, we sought to construct scalable models suitable for real-world clinical implementation. We compared models using preoperative variables alone with models additionally incorporating intraoperative and early postoperative variables. Model performance was primarily evaluated using leave-one-facility-out internal-external cross-validation (IECV), with particular emphasis on discrimination, calibration, overall probabilistic performance, and clinical utility, to assess the potential role of these models in supporting early postoperative risk stratification after noncardiac surgery under general anesthesia.

## Methods

### Study Design and Data Collection

This retrospective observational multicenter study used inpatient data from three affiliated Nippon Medical School hospitals: Nippon Medical School Hospital, Nippon Medical School Chiba Hokusoh Hospital, and Nippon Medical School Musashi Kosugi Hospital. The analytic dataset was constructed by integrating diagnosis procedure combination (DPC) claims data, laboratory test results extracted from EHRs, and perioperative information obtained from operating room and anesthesia information systems routinely used at each institution. All data sources are part of standard clinical documentation and administrative workflows.

### Ethical Considerations

The study protocol was approved by the Central Ethics Committee of Nippon Medical School (approval number M-2024‐227) and was conducted in accordance with the principles of the Declaration of Helsinki. Because this was a retrospective observational study using deidentified data, informed consent was obtained through an opt-out procedure. A prespecified statistical analysis plan for the primary analyses was developed and archived before model development and statistical analysis to minimize analytic flexibility and enhance transparency. Additional sensitivity and exploratory analyses were conducted during the revision process to evaluate the robustness and outcome specificity of the prediction models. This study was reported in accordance with the TRIPOD+AI (Transparent Reporting of a Multivariable Prediction Model for Individual Prognosis or Diagnosis+Artificial Intelligence) statement and the STROBE (Strengthening the Reporting of Observational Studies in Epidemiology) guidelines [21].

### Study Population

We included all adult inpatients who underwent noncardiac surgery under general anesthesia between January 2021 and March 2025. Each surgical admission was treated as a single analytic episode. Patients were excluded if they (1) were younger than 18 years, (2) underwent emergency surgery, defined as a decision-to-incision time of ≤24 hours recorded in the operating room information system, or (3) underwent cardiovascular surgery, including cardiac, major vascular, or thoracic aortic procedures. Cardiovascular surgeries were excluded because they follow distinct perioperative pathways and risk profiles, which could introduce substantial heterogeneity and impair model generalizability.

### Outcomes

The primary end point was a composite outcome defined a priori, capturing clinically meaningful complications occurring within 30 days after surgery. The composite comprised (1) in-hospital death, (2) unplanned reoperation, (3) prolonged mechanical ventilation (≥48 hours of invasive support or reintubation within 48 hours), (4) new initiation of renal replacement therapy, and (5) postoperative infection. Although these events reflect heterogeneous pathophysiological mechanisms, they were combined to represent major postoperative morbidity with substantial clinical consequences. This composite outcome structure has been widely adopted in large-scale perioperative risk prediction studies to improve statistical power while maintaining clinical relevance [5,22,23]. Unplanned reoperation was defined as any emergency surgical procedure performed within 30 days of the index operation. Postoperative infection was defined according to the PERISCOPE study framework as administration of systemic antibiotics for 72 hours or longer after surgery, excluding prophylactic use [24].

All outcome components were ascertained from structured EHR fields using prespecified operational definitions. Nonfatal cardiac complications were not included in the composite outcome because the end point was designed to capture major postoperative events that could be consistently ascertained across participating hospitals using routinely collected clinical data. In particular, postoperative myocardial injury could not be uniformly ascertained because serial postoperative cardiac troponin measurements were not systematically performed in all patients across the participating institutions. To assess the influence of outcome composition and the predominance of postoperative infection, we conducted a prespecified sensitivity analysis using postoperative infection as a standalone outcome and a post hoc sensitivity analysis using a composite end point excluding postoperative infection. The latter comprised in-hospital death, unplanned reoperation, new renal replacement therapy, and prolonged postoperative mechanical ventilation. Patients with postoperative infection remained eligible for this analysis and were classified as having the noninfection composite outcome if they experienced at least one of the remaining components.

### Variables

The DPC data included demographic characteristics (age and sex), anthropometric measurements (height, weight, and BMI; kg/m²), and clinical information such as comorbidities, procedures, and medications [25]. High-risk surgical procedures were defined based on the Japanese Circulation Society 2022 Guideline on Perioperative Cardiovascular Assessment and Management for Non-Cardiac Surgery [26]. Procedures were classified as high risk if the estimated 30-day risk of cardiac death or nonfatal acute myocardial infarction exceeded 5%. These included pneumonectomy and lobectomy, lung and liver transplantation, esophagectomy, pancreaticoduodenectomy, hepatectomy and biliary tract surgery, surgery for gastrointestinal perforation, total cystectomy, major lower-limb amputation, and adrenalectomy. This classification was used solely to define a preoperative predictor and was independent of the definition of the study outcome.

Drug categories were defined according to previously reported associations with postoperative complications. Sex was encoded as a binary variable (female=0, male=1) and included as a predictor in both feature sets.

Preoperative laboratory and medication variables were extracted from the 60-day period before surgery. For laboratory tests, the value recorded closest to the admission date within this window was selected. From the operating room information system, we retrieved the ASA-PS, operative duration, fluid balance, and intraoperative transfusion volumes, including red blood cells (RBCs), platelets, and fresh frozen plasma (FFP). In addition, heart rate and mean arterial pressure measured 6 hours after surgery were collected as indicators of early postoperative physiological status.

Two prespecified feature sets were constructed. The preoperative feature set comprised 36 predictors, including demographic and anthropometric characteristics, ASA-PS, comorbidities, laboratory values, medication use, surgical procedure categories, number of resected sites, and the high-risk procedure indicator. The perioperative feature set comprised all 36 preoperative predictors plus 7 additional variables obtained during surgery or the early postoperative period: operative time, RBC transfusion volume, FFP transfusion volume, platelet transfusion volume, intraoperative fluid balance, heart rate 6 hours after surgery, and mean arterial pressure 6 hours after surgery. A complete feature dictionary, including the definition, unit or coding, measurement window, missingness, preprocessing procedure, and inclusion of each predictor in the preoperative and perioperative feature sets, is provided in Table S1 in Multimedia Appendix 1.

Continuous intraoperative waveform data were not included because standardized high-resolution signals were unavailable across institutions, and single-point summary statistics derived from waveforms were considered unreliable for model training. Instead, routinely available perioperative variables were used to ensure consistency, reproducibility, and scalability across sites.

### Data Cleaning and Imputation

Continuous variables were winsorized at the 0.5th and 99.5th percentiles to limit the influence of extreme values while preserving the overall data distribution. Within each hold-out, IECV, calibration, and hyperparameter-tuning split, the winsorization thresholds were estimated using the corresponding development or training data and applied unchanged to the validation data. Variables were transformed using the natural logarithm of (1+x) when all observed values after winsorization were nonnegative and their absolute skewness exceeded 1. After imputation, continuous, ordinal, and count predictors were robustly scaled for logistic regression (LR) only; no scaling was applied for extreme gradient boosting (XGB) or light gradient boosting machine (LGBM). As shown in Table S1 in Multimedia Appendix 1, the highest proportion of missing values was 10.4% (3714/35,702) for C-reactive protein, whereas all other variables had 3.1% (1098/35,702) or less missingness.

Because the missing-data mechanism was unlikely to be strictly missing completely at random, single imputation was performed using the scikit-learn IterativeImputer with LGBMRegressor [27] (n_estimators=50, max_iter=5, random_state=0) as the conditional estimator, mean initialization, and a maximum of 5 imputation iterations. Single imputation was selected to ensure computational feasibility and model stability in this large multicenter data set. To evaluate the robustness of the findings to imputation uncertainty, we conducted a multiple-imputation sensitivity analysis using 10 stochastic imputations. The imputations were generated using the scikit-learn IterativeImputer with BayesianRidge as the conditional estimator and posterior sampling enabled (sample_posterior=True). Mean imputation was used only for initialization; the maximum number of iterations was 5, and 20 nearest features were used for each conditional imputation model. Within each IECV fold, the imputer and all data-dependent preprocessing procedures were fitted using the development facilities only and were subsequently applied to the held-out facility without reestimation. The assumption that data were missing at random may not fully hold, particularly for laboratory tests, and potential residual bias is therefore acknowledged.

### Model Development

We developed predictive models using 3 classification algorithms: LR, XGB [28], and LGBM. These algorithms were selected to represent a spectrum from conventional regression-based modeling to nonlinear tree-based ensemble methods with increasing model complexity.

Two model variants were constructed: (1) the preoperative model, which included only variables available before surgery, and (2) the perioperative model, which included preoperative, intraoperative, and early postoperative variables (within 6 hours).

Leave-one-facility-out IECV was designated as the primary validation framework. In each IECV iteration, models were developed using data from 2 institutions and evaluated in the remaining institution. This process was repeated until each institution had served once as the held-out validation institution. The out-of-sample predictions from the 3 held-out institutions were pooled to estimate overall IECV performance. A stratified random 80:20 hold-out analysis was conducted as a secondary validation.

Hyperparameter optimization for all models was conducted separately within each development data set using Optuna (Preferred Networks, Inc.) with a tree-structured Parzen estimator sampler. The optimization objective was the average precision score calculated from out-of-fold predictions generated by 5-fold stratified cross-validation. To address class imbalance of the composite outcome (event rate 10.9%, 3884/35,702), the optimization process included algorithm-specific imbalance handling: class weights for LR and scale_pos_weight for XGB and LGBM. The hyperparameter search spaces, optimization budgets, and selected values are summarized in Table S2 in Multimedia Appendix 1.

All model development steps, including preprocessing, hyperparameter optimization, model fitting, and probability calibration, were restricted to the corresponding development data. Probability calibration was performed using isotonic regression fitted to out-of-fold predictions generated by 3-fold stratified cross-validation within the corresponding development data [29]. The fitted calibration procedure was subsequently applied to the corresponding held-out data without recalibration using validation data.

### Model Evaluation

Model performance was evaluated using 2 complementary validation frameworks. The primary validation used leave-one-facility-out IECV across the 3 participating institutions: Facility A, Nippon Medical School Hospital; Facility B, Nippon Medical School Chiba Hokusoh Hospital; and Facility C, Nippon Medical School Musashi Kosugi Hospital. In each of 3 validation rounds, data from 2 facilities were used for model development, and data from the remaining facility were reserved for held-out validation. All preprocessing, hyperparameter optimization, and probability calibration procedures were performed separately within each round using the development facilities only. Facility-specific performance was evaluated in each held-out facility, and the held-out predictions from all 3 rounds were pooled for the primary overall assessment. IECV was interpreted as an assessment of model transportability across hospitals within the same academic network. A fixed 80:20 random hold-out split, stratified by the primary outcome, was used as a complementary secondary validation.

Discrimination was assessed using the area under the receiver operating characteristic curve (AUROC) and the area under the precision-recall curve (PR-AUC). Overall prediction accuracy was assessed using the Brier score and the scaled Brier score, calculated relative to a null model based on outcome prevalence. Calibration was evaluated using the calibration intercept, calibration slope, and decile-based calibration plots. Within each validation framework, isotonic calibration was fitted exclusively within the corresponding development data and subsequently applied to the held-out facility or random hold-out set without recalibration using validation data. Thus, the reported calibration metrics represent out-of-sample performance after application of a calibration procedure estimated without using validation data.

Clinical utility was assessed using decision curve analysis based on facility-specific and pooled held-out IECV predictions over threshold probabilities ranging from 0.01 to 0.30. For this exploratory analysis, a positive prediction was interpreted as prompting prioritized postoperative clinical reassessment rather than a prespecified intervention. Such reassessment could include evaluation of the patient’s recovery trajectory, organ function, and evolving clinical findings, followed by monitoring, diagnostic testing, or treatment as clinically indicated. A negative prediction indicated no model-triggered reassessment beyond usual care. The treat-all and treat-none strategies represented prioritized reassessment of all patients and no model-triggered reassessment, respectively. Because no single intervention corresponded to all components of the composite outcome, the decision curve analysis was considered exploratory.

Bootstrapping was performed on the evaluation data only, with model predictions held fixed, for the random hold-out test set, each held-out IECV facility, and the pooled held-out IECV predictions. Accordingly, these CIs reflect uncertainty arising from the evaluation sample and do not incorporate variability from model development.

### Sensitivity Analyses

Sensitivity analyses addressed 3 domains. First, to assess the robustness of the findings to uncertainty in missing-value imputation, a multiple-imputation sensitivity analysis was conducted for the preoperative and perioperative LightGBM models using 10 stochastically imputed data sets. This analysis was restricted to the primary composite outcome and used leave-one-facility-out IECV. The facility-specific LGBM hyperparameters selected in the primary analysis were held fixed. Within each imputed data set and IECV fold, the model was refitted, and isotonic calibration was estimated from out-of-fold predictions generated by 3-fold stratified cross-validation within the development data. Patient-level calibrated probabilities were averaged across the 10 imputed data sets before performance was evaluated using the pooled held-out predictions. Second, to assess the influence of outcome composition, preoperative and perioperative LGBM models were refitted using postoperative infection as the sole outcome and, in an additional post hoc analysis, using a composite end point excluding the postoperative infection component. These alternative-outcome analyses were evaluated using leave-one-facility-out IECV, with held-out predictions pooled across the 3 facilities. Third, temporal validation was performed by developing the LR, XGBoost, and LGBM models using data from January 2021 through December 2023 and evaluating them without model updating in a temporally held-out cohort from January 2024 through March 2025. In all sensitivity analyses, data-dependent preprocessing procedures were fitted using the corresponding development data only and applied unchanged to the held-out data. For the alternative-outcome analyses, the LGBM hyperparameters selected for the primary outcome were held fixed, with scale_pos_weight recalculated within each corresponding development data set. For temporal validation, hyperparameter optimization and probability calibration were performed using the temporal development cohort only.

### Model Interpretation

Model predictions were interpreted using Shapley additive explanations (SHAP) values [30].

SHAP values were calculated separately for out-of-sample observations from each held-out institution and subsequently pooled across the 3 IECV folds. To limit computational burden while preserving the facility-specific outcome distribution, up to 2000 patients were sampled from each held-out institution using stratified random sampling by outcome status. LR models were interpreted using a linear SHAP explainer, whereas XGB and LGBM models were interpreted using a tree-based SHAP explainer. SHAP values were calculated for the uncalibrated base-model output because isotonic regression was applied as a separate postmodel calibration step. Accordingly, SHAP values were expressed on the log-odds scale for LR and on the raw margin scale for the tree-based models. Global SHAP summary plots were used to examine the magnitude and direction of individual predictor contributions.

Feature interactions were evaluated for the XGB and LGBM models using pairwise SHAP interaction values. Absolute interaction values were averaged across the sampled pooled out-of-sample observations, and diagonal elements representing main effects were set to 0. For visualization, values below the within-model 80th percentile were masked, and the 15 predictors with the greatest total interaction strength were displayed. SHAP values and interaction values represent model-based predictive contributions and were not interpreted as causal effects or compared directly in magnitude across algorithms.

### Statistical Analysis

Continuous variables are reported as median (25th, 75th percentile), and categorical variables as number (percentage). For comparisons across the 3 institutions, standardized mean differences (SMD) were calculated for each pair of institutions and summarized as the mean absolute pairwise SMD. Values below 0.10 were considered to indicate limited between-institution imbalance. The 95% CIs for individual model-performance estimates were calculated using 1000 stratified bootstrap resamples of the corresponding validation sample, with model predictions held fixed. Differences in AUROC between the preoperative and perioperative models were evaluated using the paired DeLong method. Differences in PR-AUC were evaluated using paired bootstrap resampling stratified by held-out institution and outcome status. Percentile 95% CIs were obtained from 10,000 bootstrap resamples, and 2-sided *P* values were calculated from the null-centered bootstrap distribution of the paired difference. Between-algorithm differences were not formally tested. *P* values were interpreted descriptively and were not adjusted for multiple comparisons.

Statistical analyses were performed using R version 4.4.1 (R Foundation for Statistical Computing), and ML analyses were conducted using Python version 3.11.11 (Python Software Foundation).

## Results

### Study Population and Baseline Characteristics

We identified 45,790 patients who underwent surgery under general anesthesia at 3 facilities between January 2021 and March 2025. After excluding patients aged younger than 18 years (n=2918), those undergoing emergency surgery (n=5176), and those undergoing cardiac surgery (n=1994), 35,702 patients were included in the final analytic cohort. The cohort comprised 20,304 patients from Facility A, 9454 from Facility B, and 5944 from Facility C (Figure 1).

**Figure 1.**
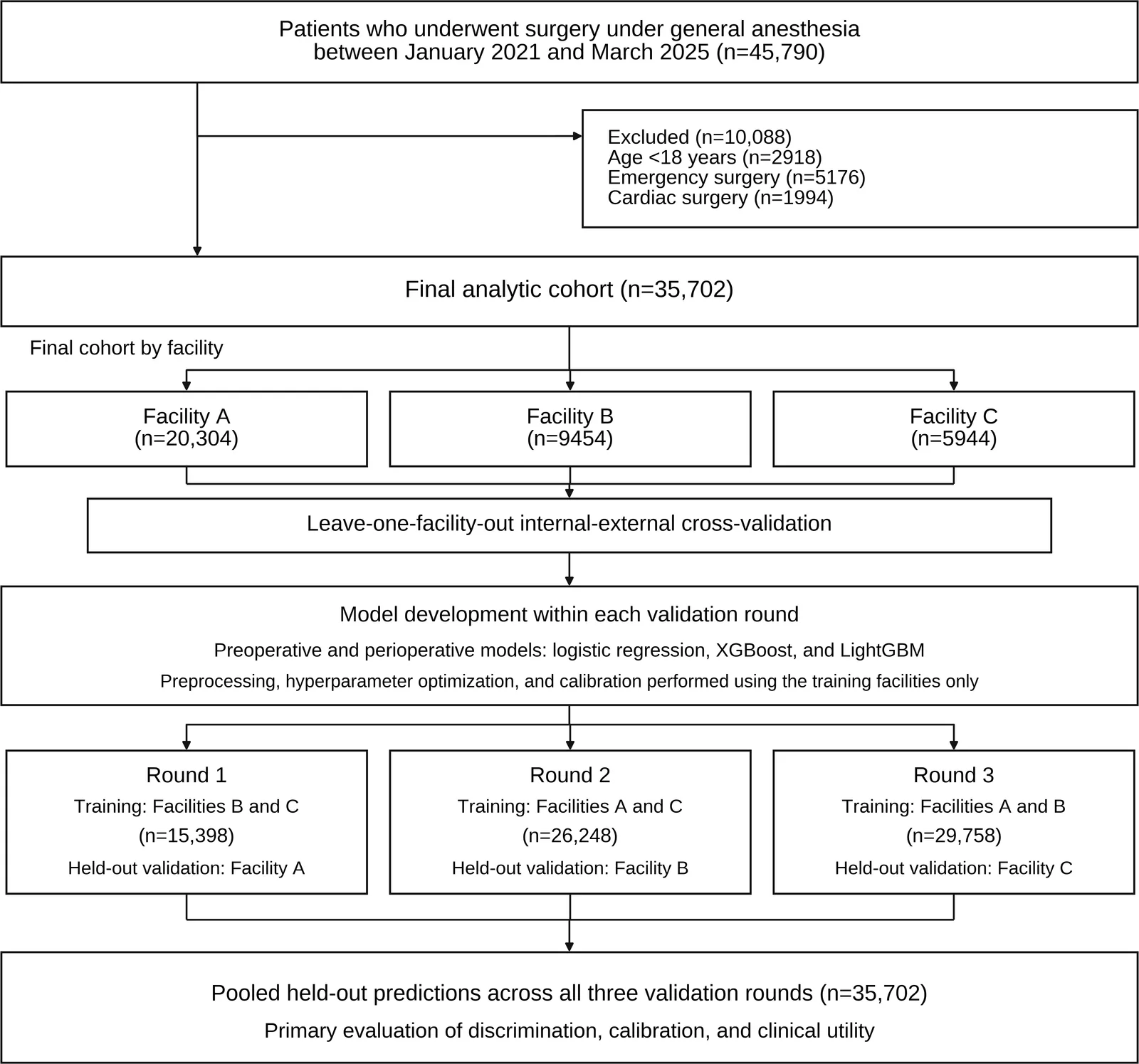
Study cohort selection and leave-one-facility-out internal-external cross-validation framework.

After applying the exclusion criteria, 35,702 patients from 3 facilities were included in the final analytic cohort. In each validation round, 2 facilities were used for model development, and the remaining facility was reserved for held-out validation. Preprocessing, hyperparameter optimization, and calibration were performed separately within each round using the training facilities only. Held-out predictions from the 3 validation rounds were pooled for the primary evaluation of discrimination, calibration, and clinical utility.

Table 1 summarizes the selected characteristics and outcome prevalence of the 35,702 surgical admissions included in the study. Of these, 20,304 (56.9%) were from Facility A, 9454 (26.5%) from Facility B, and 5944 (16.6%) from Facility C. The median age was 64 years (IQR 49‐75), 17,494 patients (49.0%) were male, and the median BMI was 23.0 kg/m² (IQR 20.6‐25.6). Overall, 30,325 patients (84.9%) were classified as ASA-PS I or II.

**Table 1.** Selected characteristics and outcome prevalence of the study cohort overall and by facility.^a^

Variable	Overall (N=35,702)	Facility A (N=20,304)	Facility B (N=9454)	Facility C (N=5944)	SMD^b,c^
Outcomes, n (%)					
Composite end point^d^	3884 (10.9)	2191 (10.8)	1115 (11.8)	578 (9.7)	0.045
Composite end point excluding the postoperative infection component^e^	1061 (3.0)	652 (3.2)	281 (3.0)	128 (2.2)	0.044
Postoperative infection^f^	3473 (9.7)	1939 (9.5)	1006 (10.6)	528 (8.9)	0.040
Demographic characteristics					
Age, median (IQR), years	64 (49-75)	64 (49-75)	68 (53-75)	58 (44-73)	0.249
Male sex, n (%)	17,494 (49.0)	9562 (47.1)	5123 (54.2)	2809 (47.3)	0.095
BMI, median (IQR), kg/m²	23.0 (20.6‐25.6)	22.9 (20.5‐25.6)	23.3 (21.0‐26.0)	22.5 (20.2‐25.1)	0.114
ASA^g^ physical status, n (%)					0.179
Class I	4428 (12.4)	2333 (11.5)	1127 (11.9)	968 (16.3)	
Class II	25,897 (72.5)	14,531 (71.6)	6962 (73.6)	4404 (74.1)	
Class III	5254 (14.7)	3328 (16.4)	1359 (14.4)	567 (9.5)	
Class IV	123 (0.3)	112 (0.6)	6 (0.1)	5 (0.1)	
Preoperative laboratory values, median (IQR)					
Hemoglobin, g/dL	13.2 (12.1‐14.3)	13.2 (12.0‐14.2)	13.4 (12.2‐14.5)	13.4 (12.2‐14.4)	0.075
Albumin, g/dL	4.2 (3.9‐4.4)	4.2 (3.9‐4.5)	4.2 (3.9‐4.4)	4.2 (3.9‐4.5)	0.065
Creatinine, mg/dL	0.77 (0.64‐0.93)	0.76 (0.63‐0.93)	0.79 (0.65‐0.95)	0.77 (0.65‐0.93)	0.014
Preoperative medications, n (%)					
Diabetes medications	5000 (14.0)	2805 (13.8)	1592 (16.8)	603 (10.1)	0.131
Anticoagulants	1816 (5.1)	1048 (5.2)	537 (5.7)	231 (3.9)	0.056
Antiplatelet agents	1861 (5.2)	1008 (5.0)	597 (6.3)	256 (4.3)	0.060
Antineoplastic agents	2610 (7.3)	1701 (8.4)	551 (5.8)	358 (6.0)	0.066
Intraoperative variables, median (IQR)					
Operative duration, min	132 (83-207)	137 (89-209)	125 (76-215)	122 (78-182)	0.098
Fluid balance, mL	949 (618-1,541)	1,050 (700-1,781)	785 (500-1,336)	855 (625-1,190)	0.314
Early postoperative physiological variables within 6 hours after surgery, median (IQR)					
Heart rate, beats/min	75 (67-85)	76 (68-86)	75 (67-85)	72 (64-82)	0.173
Mean arterial pressure, mm Hg	88 (79-98)	89 (80-98)	87 (79-97)	88 (78-98)	0.088

a Data are presented as median IQR or n (%). Statistics were based on observed data before imputation. Variable definitions and missingness are provided in Supplementary Table S1 in Multimedia Appendix 2.

b SMD: standardized mean difference.

c SMDs are the mean absolute pairwise values across facilities.

d The composite end point comprised in-hospital death, unplanned reoperation, prolonged mechanical ventilation, new initiation of renal replacement therapy, and postoperative infection.

e The composite end point excluding postoperative infection comprised the remaining components.

f Patients with postoperative infection were included if they also experienced any of these components.

g ASA: American Society of Anesthesiologists.

The primary composite end point occurred in 3884 of 35,702 patients (10.9%). Postoperative infection occurred in 3473 patients (9.7%), whereas the composite end point excluding postoperative infection occurred in 1061 patients (3.0%). Outcome prevalence was similar across facilities. The prevalence of the primary composite end point ranged from 9.7% (578/5944) to 11.8% (1115/9454), postoperative infection from 8.9% (528/5944) to 10.6%(1006/9454), and the composite end point excluding postoperative infection from 2.2% (128/5944) to 3.2% (652/20,304); the corresponding SMDs were all 0.045 or lower.

In contrast, differences in case mix and perioperative characteristics were observed across facilities. The largest between-facility differences were identified for intraoperative net fluid balance (SMD 0.314), surgical procedure category (maximum SMD 0.290), age (SMD 0.249), ASA-PS (SMD 0.179), and heart rate at 6 hours after surgery (SMD 0.173). Patients at Facility B were older, whereas those at Facility C were younger and more frequently classified as ASA-PS I. Facility A had a higher median intraoperative net fluid balance than Facilities B and C. Most preoperative laboratory measurements showed limited variation across facilities. Complete facility-specific characteristics are provided in Table S3 in Multimedia Appendix 2.

In the secondary random hold-out analysis, outcome prevalence was similar between the development and hold-out test sets. In the temporal validation analysis, event rates and most baseline and perioperative characteristics were also comparable between periods, although modest differences were observed for serum sodium and potassium. Detailed cohort characteristics are provided in Tables S4 and S5 in Multimedia Appendix 2.

The distribution of transfused blood products is shown in Figure S1 in Multimedia Appendix 3, with median transfusion volumes of 4 units (IQR 2‐6) for FFP, 10 units (IQR 8‐16) for platelets, and 2 units (IQR 1‐4) for RBCs.

### Evaluation of Prediction Models

Using pooled out-of-sample predictions from leave-one-facility-out IECV, the perioperative models consistently demonstrated better discrimination than the corresponding preoperative models (Tables 2 and 3 and Figure 2). AUROC increased from 0.784‐0.791 for the preoperative models to 0.810‐0.817 for the perioperative models, while PR-AUC increased from 0.356‐0.361 to 0.405‐0.413. The increases in AUROC were 0.025 (95% CI 0.021‐0.030) for LR, 0.026 (95% CI 0.021‐0.030) for XGB, and 0.025 (95% CI 0.021‐0.030) for LGBM. The corresponding increases in PR-AUC were 0.053 (95% CI 0.043‐0.062), 0.055 (95% CI 0.045‐0.065), and 0.049 (95% CI 0.039‐0.058), respectively (all *P*<.001).

**Table 2.** Performance of preoperative and perioperative prediction models based on pooled out-of-sample predictions from internal-external cross-validation.

Model and feature set	AUROC^a^ (95% CI)	Difference in AUROC (95% CI)^b^	PR-AUC^c^ (95% CI)	Difference in PR-AUC (95% CI)^b^
Logistic regression				
Preoperative	0.784 (0.777‐0.793)	—^d^	0.361 (0.347‐0.377)	—
Perioperative	0.810 (0.802‐0.817)	+0.025 (0.021‐0.030)	0.413 (0.399‐0.429)	+0.053 (0.043‐0.062)
XGB^e^				
Preoperative	0.791 (0.783‐0.799)	—	0.358 (0.344‐0.373)	—
Perioperative	0.817 (0.809‐0.824)	+0.026 (0.021‐0.030)	0.413 (0.398‐0.429)	+0.055 (0.045‐0.065)
LGBM^f^				
Preoperative	0.788 (0.780‐0.796)	—	0.356 (0.342‐0.370)	—
Perioperative	0.813 (0.805‐0.820)	+0.025 (0.021‐0.030)	0.405 (0.389‐0.419)	+0.049 (0.039‐0.058)

a AUROC: area under the receiver operating characteristic curve.

b *P*<.001

c PR-AUC: area under the precision-recall curve.

d Not applicable.

e XGB: extreme gradient boosting.

f LGBM: light gradient boosting machine.

**Table 3.** Overall probabilistic performance and calibration.

Model and feature set	Brier score (95% CI)	Scaled Brier score (95% CI)	Calibration slope (95% CI)	Calibration intercept (95% CI)
Logistic regression				
Preoperative	0.082 (0.081 to 0.083)	0.151 (0.141 to 0.163)	0.921 (0.887 to 0.960)	−0.144 (−0.205 to −0.075)
Perioperative	0.078 (0.077 to 0.080)	0.191 (0.179 to 0.203)	0.899 (0.868 to 0.928)	−0.222 (−0.272 to −0.170)
XGB^a^				
Preoperative	0.083 (0.081 to 0.084)	0.148 (0.136 to 0.160)	0.885 (0.853 to 0.919)	−0.237 (−0.292 to −0.180)
Perioperative	0.079 (0.078 to 0.080)	0.187 (0.174 to 0.199)	0.875 (0.840 to 0.908)	−0.301 (−0.357 to −0.250)
LGBM^b^				
Preoperative	0.083 (0.082 to 0.084)	0.144 (0.133 to 0.155)	0.913 (0.882 to 0.948)	−0.287 (−0.339 to −0.232)
Perioperative	0.080 (0.079 to 0.081)	0.175 (0.162 to 0.187)	0.902 (0.870 to 0.932)	−0.367 (−0.415 to −0.319)

a XGB: extreme gradient boosting.

b LGBM: light gradient boosting machine.

**Figure 2.**
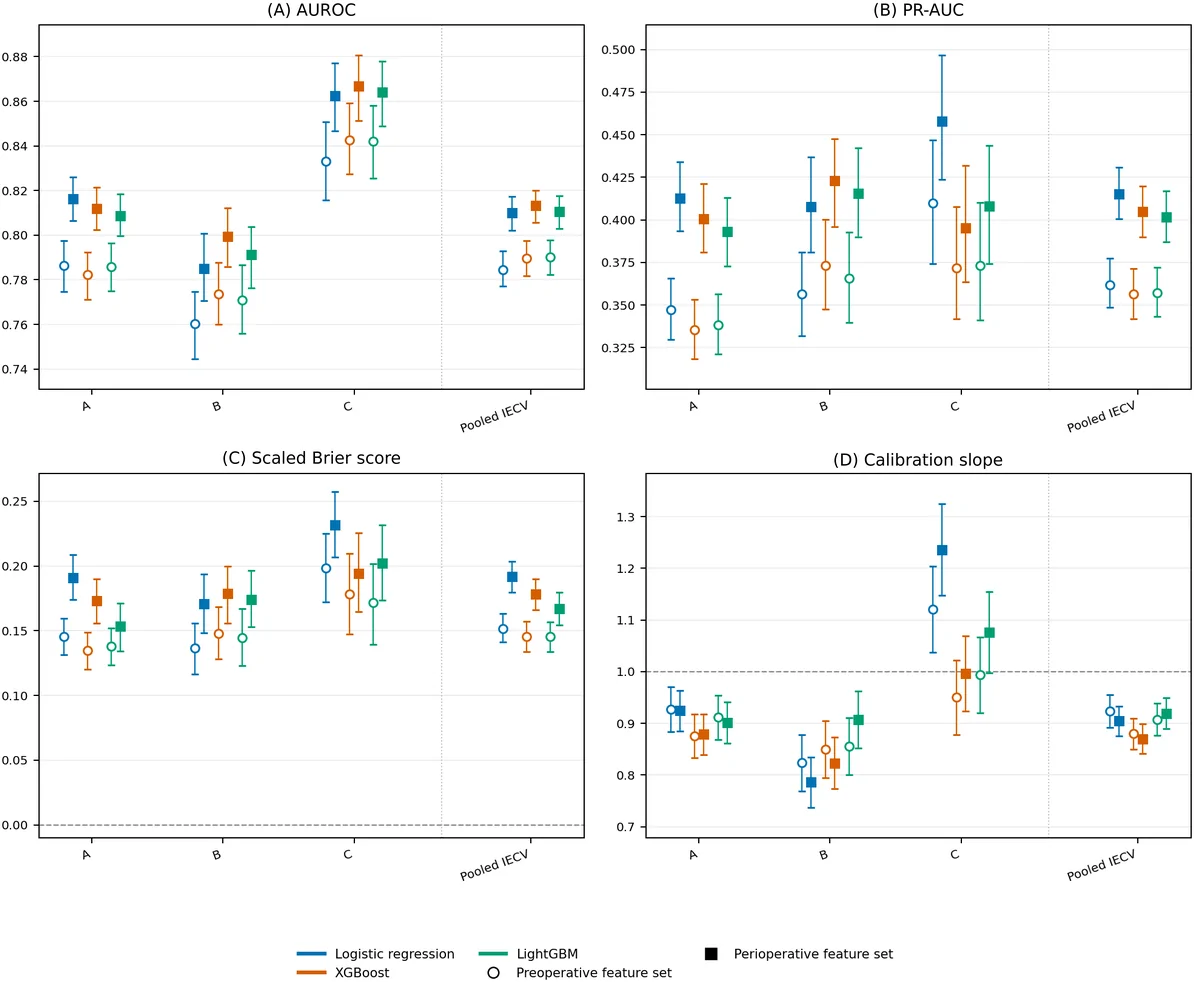
Performance of preoperative and perioperative prediction models in internal-external cross-validation (IECV). Facility-specific estimates were calculated from predictions for each held-out facility, and pooled internal-external cross-validation estimates were calculated by combining all out-of-sample predictions. The panels show (A) the area under the receiver operating characteristic curve (AUROC), (B) the area under the precision-recall curve, calculated using average precision (PR-AUC), (C) the scaled Brier score, and (D) the calibration slope. Filled squares indicate models using the perioperative feature set, and open circles indicate models using the preoperative feature set. Blue, orange, and green markers represent logistic regression (LR), extreme gradient boosting (XGB), and light gradient boosting machine (LGBM), respectively. Error bars indicate 95% CIs. The vertical dotted line separates facility-specific results from the pooled estimates. The horizontal reference lines indicate a scaled Brier score of 0, corresponding to a prevalence-based null model, and the ideal calibration slope of 1. Higher values indicate better performance for the discrimination metrics and scaled Brier score, whereas calibration slopes closer to 1 indicate better calibration.

In Tables 2 and 3, values are based on pooled out-of-sample predictions obtained from leave-one-facility-out IECV. In each fold, models were developed and calibrated using data from 2 institutions and evaluated in the remaining institution without model updating. The pooled analysis included 35,702 surgical admissions and 3884 primary composite end point events. Differences were calculated as the performance of the perioperative model minus that of the preoperative model within the same algorithm. A PR-AUC was calculated using average precision. The 95% CIs for individual performance estimates were calculated using 1000 stratified bootstrap resamples. Differences in AUROC and their 95% CIs were evaluated using the paired DeLong method. Differences in PR-AUC were evaluated using paired bootstrap resampling stratified by held-out institution and outcome status. Percentile 95% CIs were calculated from 10,000 bootstrap resamples, and 2-sided *P* values were obtained from the null-centered bootstrap distribution of the paired difference. *P* values were interpreted descriptively and were not adjusted for multiple comparisons. Between-algorithm differences were not formally tested because the prespecified comparison concerned the incremental value of perioperative predictors within each algorithm. Lower Brier scores and higher scaled Brier scores indicate better overall probabilistic performance. A scaled Brier score of 0 indicates performance equivalent to a prevalence-based null model, whereas a value of 1 indicates perfect prediction. The ideal values for the calibration slope and calibration intercept are 1 and 0, respectively.

The addition of perioperative predictors also improved overall probabilistic performance. Brier scores decreased from 0.082‐0.083 to 0.078‐0.080, whereas scaled Brier scores increased from 0.144‐0.151 to 0.175‐0.191. However, calibration remained imperfect. The pooled calibration slopes of the perioperative models ranged from 0.875 to 0.902, and calibration intercepts ranged from −0.367 to −0.222, indicating that the predicted probabilities were somewhat too extreme and tended to overestimate the overall event risk.

The facility-specific IECV results were directionally consistent, although performance and calibration varied across the held-out institutions (Table S6 in Multimedia Appendix 2 and Figure S2 in Multimedia Appendix 3). The perioperative models had higher AUROCs at all 3 institutions. Improvements in PR-AUC were more apparent at Facilities A and B and were smaller or dependent on the algorithm at Facility C. Calibration intercepts were generally negative at Facilities A and B. At Facility C, the perioperative XGB and LGBM models were relatively close to ideal calibration, whereas the perioperative LR model showed greater deviation, with a calibration slope of 1.238 and an intercept of 0.582.

Decision curve analysis based on the IECV predictions showed that all models provided greater net benefit than the treat-none strategy across the evaluated threshold probability range of 0.01‐0.30 (Figure 3). The perioperative models generally yielded higher net benefit than the corresponding preoperative models, particularly at thresholds above approximately 0.05, although the curves overlapped substantially and differences between algorithms were small. This pattern was consistent in the pooled IECV analysis and across the 3 held-out facilities. The treat-all strategy provided lower net benefit than the prediction models and became unfavorable at thresholds near or above the observed event prevalence.

**Figure 3.**
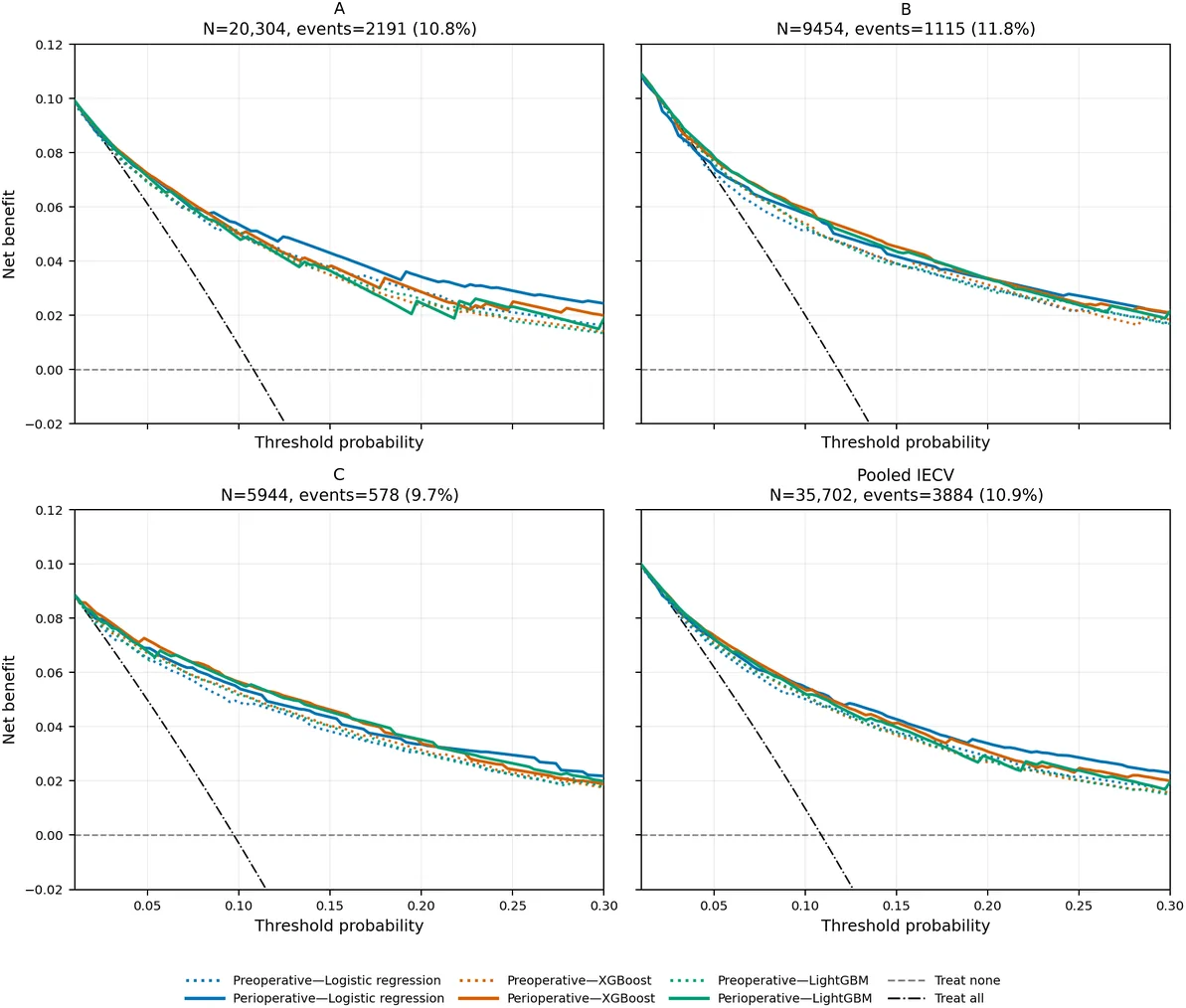
Decision curve analysis of preoperative and perioperative prediction models based on internal-external cross-validation (IECV). Net benefit is shown across threshold probabilities from 0.01 to 0.30 for each held-out facility and for pooled out-of-sample predictions. Solid lines represent perioperative models, and dotted lines represent preoperative models. The dashed gray line indicates the treat-none strategy, and the black dash-dotted line indicates the treat-all strategy. LGBM: light gradient boosting machine; XGB: extreme gradient boosting.

### Secondary and Sensitivity Analyses

The secondary random hold-out validation yielded results consistent with the primary IECV analysis (Table S7 in Multimedia Appendix 2). Across algorithms, AUROC increased from 0.787‐0.804 with the preoperative feature set to 0.808‐0.825 with the perioperative feature set, and PR-AUC increased from 0.363‐0.395 to 0.410‐0.450. Perioperative models also had lower Brier scores and higher scaled Brier scores, with calibration slopes ranging from 0.887 to 0.954.

The multiple-imputation sensitivity analysis of the LGBM models yielded results consistent with the primary IECV analysis (Table S9 in Multimedia Appendix 2). The perioperative model showed higher discrimination than the preoperative model, with an AUROC of 0.814 (95% CI 0.807‐0.821) versus 0.788 (95% CI 0.781‐0.796) and a PR-AUC of 0.413 (95% CI 0.397‐0.429) versus 0.364 (95% CI 0.350‐0.379). The perioperative model also had a lower Brier score (0.080 vs 0.083) and a higher scaled Brier score (0.178 vs 0.145). Calibration slopes were similar between the perioperative and preoperative models (0.917 and 0.919, respectively), although the calibration intercepts remained negative (−0.344 and −0.275, respectively).

In the outcome-specific analysis using LGBM, perioperative variables improved discrimination for both postoperative infection and the composite end point excluding postoperative infection (Table S8 in Multimedia Appendix 2). For the composite end point excluding postoperative infection, AUROC increased from 0.781 to 0.839 and PR-AUC from 0.173 to 0.249. For postoperative infection, AUROC increased from 0.788 to 0.809 and PR-AUC from 0.320 to 0.344. However, calibration was suboptimal for the composite end point excluding postoperative infection, particularly for the perioperative model, which had a calibration slope of 0.595 and an intercept of −1.436.

Temporal validation without model updating also supported the main findings (Table S10 in Multimedia Appendix 2). Across algorithms, perioperative models achieved AUROCs of 0.826‐0.838 and PR-AUCs of 0.424‐0.466, compared with 0.802‐0.810 and 0.374‐0.399, respectively, for the preoperative models. Calibration remained close to ideal, with slopes ranging from 1.000 to 1.048 and intercepts from −0.045 to 0.034 for the perioperative models.

### Model Interpretation

SHAP analyses showed broadly consistent patterns across LR, XGB, and LGBM models (Figure 4 and Figures S3 and S4 in Multimedia Appendix 3). In the preoperative models, serum albumin, malignancy, male sex, C-reactive protein, white blood cell count, hemoglobin, ASA-PS, and surgical procedure category were among the most influential predictors. Lower serum albumin and hemoglobin levels and higher C-reactive protein and white blood cell counts generally shifted predictions toward a higher risk of the composite end point. High-risk and neurosurgical procedures also contributed to higher predicted risk, particularly in the tree-based models.

**Figure 4.**
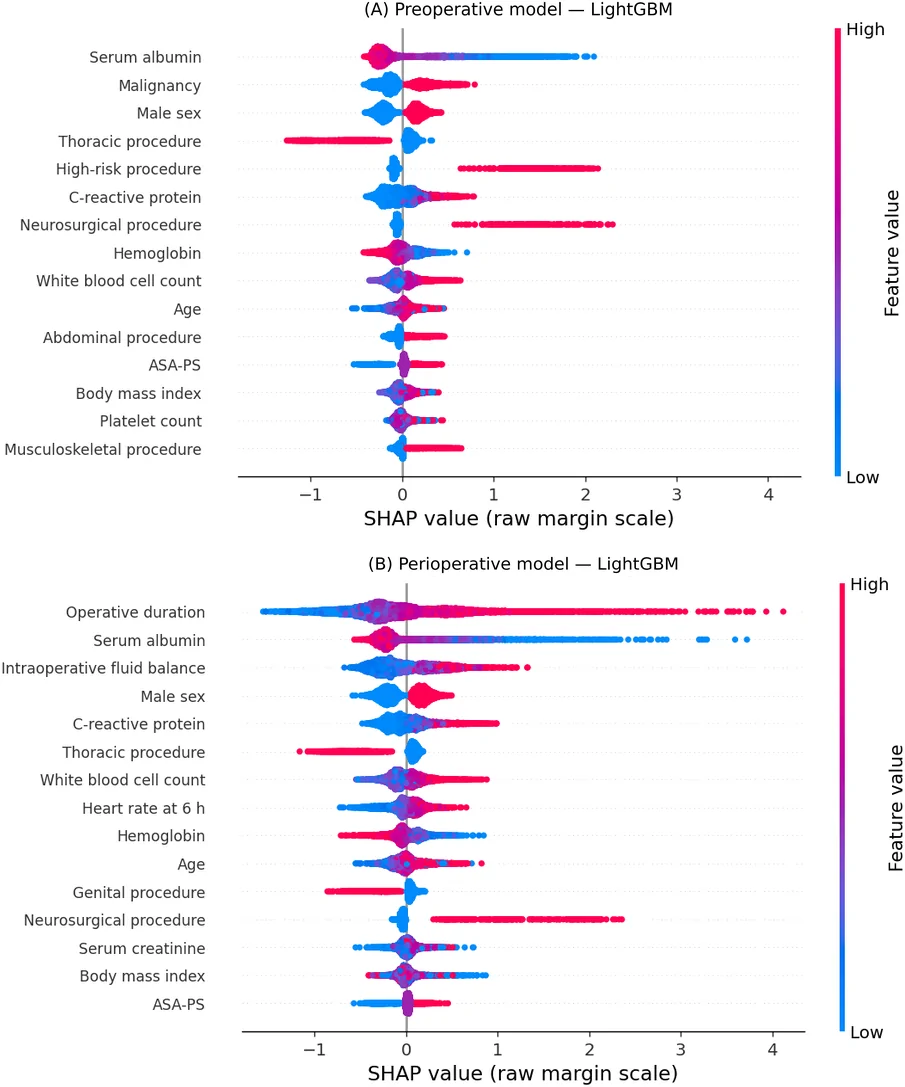
Shapley additive explanation (SHAP) summary plots for the light gradient boosting machine (LightGBM) models. (A) Preoperative model. (B) Perioperative model. Features are ranked by their mean absolute SHAP values, with the most influential features shown at the top. Each point represents 1 patient. The horizontal position indicates the contribution of the feature to the model output on the raw margin scale; positive SHAP values shift the prediction toward a higher risk of the composite end point, whereas negative values shift the prediction toward a lower risk. Point color represents the observed feature value, ranging from low (blue) to high (red). For binary variables, blue and red generally indicate absence and presence, respectively. ASA-PS: American Society of Anesthesiologists Physical Status.

After perioperative variables were added, operative duration became the most influential predictor across all 3 algorithms, with longer procedures shifting predictions toward higher risk. A larger intraoperative fluid balance and a higher heart rate at 6 hours after surgery also emerged as important predictors of higher risk. Serum albumin remained one of the leading predictors, with higher values consistently shifting predictions toward lower risk. Male sex, inflammatory markers, hemoglobin, and surgical procedure categories also remained influential. These findings indicate that the perioperative models integrated measures of operative burden and early postoperative physiology with preoperative patient vulnerability. SHAP values describe the contribution of individual features to model predictions and should not be interpreted as causal effects.

Feature interaction patterns are shown in Figure S5 in Multimedia Appendix 3. In the preoperative models, prominent interactions involved serum albumin, malignancy, inflammatory markers, and surgical procedure categories. After perioperative variables were added, operative duration emerged as the central interaction feature in both XGB and LGBM, with interactions involving intraoperative fluid balance, serum albumin, age, C-reactive protein, and surgical procedure category. Early postoperative physiological variables, including heart rate and mean arterial pressure at 6 hours, also contributed to the interaction structure.

## Discussion

### Principal Findings

This multicenter study showed that models incorporating routinely available intraoperative and early postoperative data consistently improved predictive performance over preoperative-only models in the primary IECV analysis. Across all 3 algorithms, perioperative models achieved modest but reproducible improvements in discrimination, with AUROC increasing from 0.784‐0.791 to 0.810‐0.817 and PR-AUC increasing from 0.356‐0.361 to 0.405‐0.413. Overall probabilistic performance also improved, as reflected by lower Brier scores and higher scaled Brier scores. Decision curve analysis showed an incremental net benefit of the perioperative models across the evaluated threshold range, although the substantial overlap between curves suggested that the magnitude of improvement was limited. These findings were directionally consistent in the random hold-out validation, temporal validation, and outcome-specific sensitivity analyses. However, calibration varied across institutions, and the pooled perioperative models had calibration slopes below 1 and negative calibration intercepts, indicating predictions that were somewhat too extreme and tended to overestimate risk. Taken together, these findings support the incremental value of routinely collected perioperative information for postoperative risk stratification, while indicating that local recalibration may be required to ensure accurate absolute risk estimation across institutions.

Our findings corroborate and extend prior work on perioperative risk prediction. The PERISCOPE study used gradient boosting to predict postoperative infection (AUROC 0.82) but required site-specific intercept adjustment before deployment [24]. Zhuang et al [31] developed parsimonious preoperative models across more than 30,000 surgical cases (AUROC 0.73‐0.89), likewise emphasizing the need for local recalibration. The MySurgeryRisk (University of Florida) algorithm, trained on nearly 500,000 cases from 8 US hospitals, achieved AUROCs of 0.79‐0.92 but demonstrated intersite calibration error of approximately 0.15, prompting isotonic recalibration [22]. Similarly, DeepSurv-OR, a transformer-based intraoperative model, reached an AUROC of 0.90 yet showed greater than 10% divergence in calibration across institutions [32]. Notably, most of these studies relied on AUROC-based evaluation, which is not directly comparable to PR-AUC-based assessment in low-event-rate populations. Collectively, these studies, and our multicenter validation, highlight that even high-performing models often lose calibration after transfer, making site-specific recalibration a prerequisite for safe and reliable clinical implementation.

Among baseline factors, hypoalbuminemia was the strongest predictor, consistent with prior meta-analyses across surgical specialties [33,34]. Albumin remained highly influential after incorporating intraoperative and early postoperative variables, confirming its role as a baseline determinant of surgical stress tolerance. Previous multicenter ML studies integrating high-resolution intraoperative waveform data improved PR-AUC to approximately 0.41 [32], and models combining respiratory and hemodynamic features reached PR-AUC values of approximately 0.45 for pulmonary complications [35]. Together, these findings illustrate the complementary contribution of baseline patient characteristics, such as albumin, and intraoperative and early postoperative physiological variables to postoperative risk stratification. These findings may support the evaluation of locally calibrated thresholds for prioritizing postoperative clinical reassessment, but they do not identify a specific management action for each predicted risk level. However, because the majority of patients in this cohort were classified as ASA-PS I-II, generalizability to higher-risk populations or emergency surgical settings remains to be established.

Figure 3 shows that the perioperative models provided greater net benefit than the preoperative models across a range of threshold probabilities. In this exploratory decision curve analysis, a positive prediction represented prioritization for postoperative clinical reassessment rather than automatic initiation of intensive care unit admission, infection screening, or organ support. Accordingly, the evaluated thresholds should be interpreted as reflecting trade-offs between the number of patients selected for reassessment and the number of postoperative complications missed, rather than as prescriptive treatment thresholds. Two threshold regions were particularly informative. Lower thresholds yielded high sensitivity but selected a larger proportion of patients for reassessment, whereas intermediate thresholds reduced the number of patients selected while increasing positive predictive value. These findings illustrate the potential trade-offs associated with different thresholds; however, they do not establish an optimal threshold for clinical use. Threshold selection would require prospective evaluation incorporating the intended clinical workflow, available resources, stakeholder preferences, and the consequences of false-positive and false-negative predictions. These patterns align with the original decision curve framework proposed by Vickers and Elkin [36] and the “DCA-to-threshold-to-implementation” paradigm described by Steyerberg et al [37]. Given the observed calibration drift between hospitals, site-specific recalibration, using intercept and slope adjustment or isotonic regression [38], together with stakeholder-guided threshold selection [39] and continuous postdeployment monitoring [40], is recommended to preserve clinical utility over time.

Clinically, these perioperative ML models may support early postoperative risk stratification by identifying patients who warrant earlier or more detailed clinical reassessment. The model output is not intended to directly determine intensive care unit admission, infection screening, reintubation, renal replacement therapy, or other specific interventions. Instead, subsequent monitoring, diagnostic testing, and treatment should be selected according to the patient’s evolving clinical condition and the complication suspected. Similarly, a low predicted risk should not be used in isolation to determine postoperative ward placement or to reduce usual clinical monitoring. Because all predictors used in the current models are routinely documented in anesthesia and nursing records, integration into existing EHR dashboards is technically feasible without additional data collection. Such locally calibrated tools are intended to complement, rather than replace, clinical judgment by providing additional information for risk-informed reassessment. Their effects on workflow, resource use, clinical decisions, and patient outcomes require prospective evaluation before implementation. For broader clinical adoption, future work should prioritize prospective validation and seamless integration into anesthesia information systems. Embedding automated risk alerts within perioperative dashboards may facilitate real-time decision support while maintaining human oversight and institutional calibration to ensure safe, transparent, and sustainable implementation.

This study has several limitations. First, although IECV was performed, the three participating hospitals were tertiary referral centers within the same academic network operating under a shared DPC-based bundled payment system. Accordingly, this validation should be considered internal-external (semiexternal) rather than fully external, and model performance and calibration may differ in community hospitals or international health care systems. The observed variation in calibration across facilities also indicates that local recalibration may be required before implementation. Second, retrospective use of administrative and EHR data carries inherent risks of miscoding, residual confounding, and incomplete capture of clinical context, and excludes high-resolution intraoperative signals, such as continuous blood pressure waveforms or vasoactive drug infusion rates, that could further enhance predictive performance.

Third, missing values were imputed using an LGBM-based iterative imputation approach that assumes data are missing at random; residual bias due to nonrandom missingness cannot be excluded. Although the multiple-imputation sensitivity analysis yielded findings consistent with the primary analysis, single imputation does not account for uncertainty arising from the imputation process and may therefore underestimate uncertainty related to missing data.

Fourth, the composite end point combined heterogeneous postoperative events and was predominantly composed of postoperative infection, potentially obscuring event-specific predictor-outcome relationships. Although analyses of postoperative infection and the composite end point excluding postoperative infection supported the main findings, the small numbers of several individual components precluded the development of sufficiently stable component-specific models. Therefore, the reported performance should not be assumed to apply equally to each outcome component.

Finally, algorithmic bias related to age, frailty, or differential access to postoperative care cannot be excluded and warrants careful evaluation during real-world deployment. The models have not yet been prospectively integrated into clinical workflows, leaving real-time safety and effectiveness untested. Future studies should include broader multicenter validation, incorporation of richer physiologic data, and prospective impact assessment.

### Conclusions

In this multicenter study of adult patients undergoing noncardiac surgery under general anesthesia, perioperative models incorporating routinely available intraoperative variables and early postoperative vital signs improved prediction of major postoperative complications compared with preoperative models. Exploratory decision curve analysis suggested potential value for prioritizing postoperative clinical reassessment but did not establish utility for a specific intervention or triage decision. Institutional variation in calibration indicates that local recalibration, prospective workflow evaluation, and assessment of predefined responses to model output are required before clinical implementation.

## Supplementary material

10.2196/91315 Multimedia Appendix 1 Definitions and preprocessing of predictor variables, hyperparameter search spaces, and selected model hyperparameters.

10.2196/91315 Multimedia Appendix 2 Tables reporting cohort characteristics, validation cohort composition, and model performance across internal-external cross-validation, random hold-out validation, alternative outcome analyses, multiple imputation, and temporal validation.

10.2196/91315 Multimedia Appendix 3 Figures showing transfusion distributions, calibration plots, Shapley additive explanations (SHAP) summary plots, and pairwise SHAP interaction heatmaps.

## Abbreviations

ASA-PS: American Society of Anesthesiologists physical status
AUROC: area under the receiver operating characteristic curve
DPC: Diagnosis Procedure Combination
EHR: electronic health record
FFP: fresh frozen plasma
IECV: internal-external cross-validation
LGBM: light gradient boosting machine
LR: logistic regression
ML: machine learning
PR-AUC: area under the precision-recall curve
RBC: red blood cell
SHAP: Shapley additive explanations
SMD: standardized mean difference
STROBE: Strengthening the Reporting of Observational Studies in Epidemiology
TRIPOD+AI: Transparent Reporting of a Multivariable Prediction Model for Individual Prognosis or Diagnosis+Artificial Intelligence
XGB: extreme gradient boosting
