# Supplementary material for: Machine Learning–Based Prediction of Major Postoperative Complications After Noncardiac Surgery Under General Anesthesia Using Routinely Available Electronic Health Record Data: Retrospective Multicenter Development and Validation Study
Source: JMIR Perioper Med. 2026 Oct 21;9:e91315. doi: 10.2196/91315 (PMC13724130; doi:10.2196/91315)
Supplement: Supplementary file 1 [file periop-v9-e91315-s002.docx]

**Table S3. Detailed characteristics and outcome prevalence of the study cohort overall and by facility**

| **Variable** | **Overall (n=35,702)** | **Facility A (n=20,304)** | **Facility B (n=9,454)** | **Facility C (n=5,944)** | **SMD** |
| --- | --- | --- | --- | --- | --- |
| **Outcomes** | | | | | |
| Composite endpoint, n (%) | 3,884 (10.9) | 2,191 (10.8) | 1,115 (11.8) | 578 (9.7) | 0.045 |
| Postoperative infection, n (%) | 3,473 (9.7) | 1,939 (9.5) | 1,006 (10.6) | 528 (8.9) | 0.040 |
| Composite endpoint excluding postoperative infection, n (%) | 1,061 (3.0) | 652 (3.2) | 281 (3.0) | 128 (2.2) | 0.044 |
| In-hospital death, n (%) | 59 (0.2) | 25 (0.1) | 24 (0.3) | 10 (0.2) | 0.020 |
| Unplanned reoperation, n (%) | 416 (1.2) | 276 (1.4) | 103 (1.1) | 37 (0.6) | 0.050 |
| New renal replacement therapy, n (%) | 71 (0.2) | 47 (0.2) | 14 (0.1) | 10 (0.2) | 0.013 |
| Prolonged postoperative mechanical ventilation, n (%) | 684 (1.9) | 412 (2.0) | 182 (1.9) | 90 (1.5) | 0.026 |
| **Demographic characteristics** | | | | | |
| Age, years | 64 [49-75] | 64 [49-75] | 68 [53-75] | 58 [44-73] | 0.249 |
| Male sex, n (%) | 17,494 (49.0) | 9,562 (47.1) | 5,123 (54.2) | 2,809 (47.3) | 0.095 |
| Body mass index, kg/m² | 23.0 [20.6-25.6] | 22.9 [20.5-25.6] | 23.3 [21.0-26.0] | 22.5 [20.2-25.1] | 0.114 |
| **Preoperative clinical status** | | | | | |
| ASA Physical Status, n (%) |  |  |  |  | 0.179 |
| Class I | 4,428 (12.4) | 2,333 (11.5) | 1,127 (11.9) | 968 (16.3) |  |
| Class II | 25,897 (72.5) | 14,531 (71.6) | 6,962 (73.6) | 4,404 (74.1) |  |
| Class III | 5,254 (14.7) | 3,328 (16.4) | 1,359 (14.4) | 567 (9.5) |  |
| Class IV | 123 (0.3) | 112 (0.6) | 6 (0.1) | 5 (0.1) |  |
| **Comorbidities** | | | | | |
| Malignancy, n (%) | 13,325 (37.3) | 7,878 (38.8) | 3,389 (35.8) | 2,058 (34.6) | 0.058 |
| Congestive heart failure, n (%) | 1,605 (4.5) | 1,039 (5.1) | 354 (3.7) | 212 (3.6) | 0.051 |
| Maintenance dialysis, n (%) | 369 (1.0) | 232 (1.1) | 75 (0.8) | 62 (1.0) | 0.024 |
| **Preoperative laboratory values** | | | | | |
| White blood cell count, ×10³/µL | 5.8 [4.8-7.1] | 5.8 [4.8-7.1] | 5.9 [4.9-7.1] | 5.8 [4.8-7.0] | 0.012 |
| Hemoglobin, g/dL | 13.2 [12.1-14.3] | 13.2 [12.0-14.2] | 13.4 [12.2-14.5] | 13.4 [12.2-14.4] | 0.075 |
| Platelet count, ×10³/µL | 237 [198-281] | 237 [199-282] | 234 [195-279] | 237 [201-281] | 0.027 |
| Serum albumin, g/dL | 4.2 [3.9-4.4] | 4.2 [3.9-4.5] | 4.2 [3.9-4.4] | 4.2 [3.9-4.5] | 0.065 |
| C-reactive protein, mg/dL | 0.10 [0.04-0.30] | 0.09 [0.04-0.27] | 0.10 [0.05-0.36] | 0.11 [0.06-0.37] | 0.068 |
| Blood urea nitrogen, mg/dL | 14.4 [11.7-18.0] | 14.5 [11.6-18.0] | 15.0 [12.0-18.0] | 13.8 [11.1-17.3] | 0.099 |
| Serum creatinine, mg/dL | 0.77 [0.64-0.93] | 0.76 [0.63-0.93] | 0.79 [0.65-0.95] | 0.77 [0.65-0.93] | 0.014 |
| Serum potassium, mEq/L | 4.2 [4.0-4.4] | 4.2 [4.0-4.4] | 4.2 [3.9-4.4] | 4.2 [4.0-4.4] | 0.033 |
| Serum sodium, mEq/L | 141 [140-143] | 141 [140-143] | 141 [140-143] | 142 [140-143] | 0.092 |
| Total bilirubin, mg/dL | 0.57 [0.42-0.76] | 0.56 [0.43-0.74] | 0.60 [0.40-0.80] | 0.57 [0.43-0.77] | 0.039 |
| **Preoperative medications** | | | | | |
| Any diabetes medication, n (%) | 5,000 (14.0) | 2,805 (13.8) | 1,592 (16.8) | 603 (10.1) | 0.131 |
| Oral antidiabetic medication, n (%) | 2,996 (8.4) | 1,658 (8.2) | 938 (9.9) | 400 (6.7) | 0.077 |
| Insulin or GLP-1 receptor agonist, n (%) | 3,490 (9.8) | 2,051 (10.1) | 1,048 (11.1) | 391 (6.6) | 0.106 |
| Beta-blocker, n (%) | 3,500 (9.8) | 2,146 (10.6) | 892 (9.4) | 462 (7.8) | 0.065 |
| Oral corticosteroid, n (%) | 2,270 (6.4) | 1,303 (6.4) | 441 (4.7) | 526 (8.8) | 0.112 |
| Anticoagulant, n (%) | 1,816 (5.1) | 1,048 (5.2) | 537 (5.7) | 231 (3.9) | 0.056 |
| Antiplatelet agent, n (%) | 1,861 (5.2) | 1,008 (5.0) | 597 (6.3) | 256 (4.3) | 0.060 |
| Antineoplastic agent, n (%) | 2,610 (7.3) | 1,701 (8.4) | 551 (5.8) | 358 (6.0) | 0.066 |
| Opioid, n (%) | 707 (2.0) | 356 (1.8) | 292 (3.1) | 59 (1.0) | 0.100 |
| **Surgical procedure categories** | | | | | |
| Ophthalmic procedure, n (%) | 557 (1.6) | 362 (1.8) | 120 (1.3) | 75 (1.3) | 0.028 |
| Face or neck procedure, n (%) | 2,106 (5.9) | 1,934 (9.5) | 55 (0.6) | 117 (2.0) | 0.290 |
| Thoracic procedure, n (%) | 4,590 (12.9) | 2,663 (13.1) | 912 (9.6) | 1,015 (17.1) | 0.147 |
| Musculoskeletal procedure, n (%) | 5,819 (16.3) | 3,612 (17.8) | 1,568 (16.6) | 639 (10.8) | 0.135 |
| Otorhinolaryngologic procedure, n (%) | 2,029 (5.7) | 963 (4.7) | 524 (5.5) | 542 (9.1) | 0.116 |
| Neurosurgical procedure, n (%) | 1,383 (3.9) | 774 (3.8) | 403 (4.3) | 206 (3.5) | 0.028 |
| Genital procedure, n (%) | 4,707 (13.2) | 2,300 (11.3) | 1,720 (18.2) | 687 (11.6) | 0.130 |
| Urinary tract procedure, n (%) | 3,231 (9.0) | 1,785 (8.8) | 1,195 (12.6) | 251 (4.2) | 0.206 |
| Skin or soft-tissue procedure, n (%) | 2,665 (7.5) | 1,550 (7.6) | 444 (4.7) | 671 (11.3) | 0.164 |
| Abdominal procedure, n (%) | 9,440 (26.4) | 4,940 (24.3) | 2,602 (27.5) | 1,898 (31.9) | 0.113 |
| Number of resections, n (%) |  |  |  |  | 0.096 |
| 1 | 34,899 (97.8) | 19,744 (97.2) | 9,366 (99.1) | 5,789 (97.4) |  |
| 2 | 782 (2.2) | 542 (2.7) | 87 (0.9) | 153 (2.6) |  |
| 3 | 20 (0.1) | 17 (0.1) | 1 (0.0) | 2 (0.0) |  |
| 4 | 1 (0.0) | 1 (0.0) | 0 (0.0) | 0 (0.0) |  |
| High-risk procedure, n (%) | 2,046 (5.7) | 1,211 (6.0) | 539 (5.7) | 296 (5.0) | 0.029 |
| **Intraoperative variables** | | | | | |
| Operative duration, min | 132 [83-207] | 137 [89-209] | 125 [76-215] | 122 [78-182] | 0.098 |
| Intraoperative fluid balance, mL | 949 [618-1,541] | 1,050 [700-1,781] | 785 [500-1,336] | 855 [625-1,190] | 0.314 |
| Intravenous fluid volume, mL | 1,100 [700-1,900] | 1,200 [800-2,150] | 900 [600-1,650] | 1,050 [800-1,500] | 0.263 |
| Red blood cell transfusion, n (%) | 1,144 (3.2) | 690 (3.4) | 275 (2.9) | 179 (3.0) | 0.019 |
| Fresh frozen plasma transfusion, n (%) | 346 (1.0) | 216 (1.1) | 97 (1.0) | 33 (0.6) | 0.038 |
| Platelet transfusion, n (%) | 66 (0.2) | 45 (0.2) | 14 (0.1) | 7 (0.1) | 0.017 |
| **Early postoperative physiological variables** | | | | | |
| Heart rate at 6 hours, beats/min | 75 [67-85] | 76 [68-86] | 75 [67-85] | 72 [64-82] | 0.173 |
| Mean arterial pressure at 6 hours, mm Hg | 88 [79-98] | 89 [80-98] | 87 [79-97] | 88 [78-98] | 0.088 |

**Notes:** Data are presented as median [IQR] or n (%). Statistics were calculated from observed data before imputation.

The composite endpoint comprised in-hospital death, unplanned reoperation, new renal replacement therapy, prolonged postoperative mechanical ventilation, and postoperative infection. The composite endpoint excluding postoperative infection comprised the remaining 4 components; patients with postoperative infection were retained and classified as having the outcome if they experienced at least 1 of the remaining components.

Postoperative infection was defined as systemic antibiotic administration for 72 hours or longer after surgery, excluding prophylactic use. Prolonged postoperative mechanical ventilation was defined as invasive mechanical ventilation for 48 hours or longer or reintubation within 48 hours.

SMDs are the mean absolute pairwise standardized mean differences across the 3 facilities. For multicategory variables, the SMD is shown in the corresponding parent row.

**Abbreviations:** ASA, American Society of Anesthesiologists; GLP-1, glucagon-like peptide-1; IQR, interquartile range; SMD, standardized mean difference.

**Table S4. Summary of the secondary random hold-out split**

| **Measure** | **Development set** | **Hold-out test set** |
| --- | --- | --- |
| Surgical admissions, n | 28,561 | 7,141 |
| Composite endpoint, n (%) | 3,107 (10.9) | 777 (10.9) |
| Postoperative infection, n (%) | 2,764 (9.7) | 709 (9.9) |
| Composite endpoint excluding postoperative infection, n (%) | 871 (3.0) | 190 (2.7) |

**Note:** The full cohort was stratified by composite endpoint status and randomly divided into an 80% development set and a 20% independent hold-out test set.

**Table S5. Characteristics of the temporal development and validation cohorts**

| **Variable** | **Development cohort, January 2021–December 2023 (n=24,609)** | **Temporal validation cohort, January 2024–March 2025 (n=11,093)** | **SMD** |
| --- | --- | --- | --- |
| **Outcomes** | | | |
| Composite endpoint, n (%) | 2,694 (10.9) | 1,190 (10.7) | 0.007 |
| Postoperative infection, n (%) | 2,404 (9.8) | 1,069 (9.6) | 0.004 |
| Composite endpoint excluding postoperative infection, n (%) | 753 (3.1) | 308 (2.8) | 0.017 |
| **Demographic and preoperative status** | | | |
| Age, years | 65 [49–74] | 64 [50–75] | 0.003 |
| Male sex, n (%) | 12,038 (48.9) | 5,456 (49.2) | 0.005 |
| Body mass index, kg/m² | 23.0 [20.6–25.6] | 23.0 [20.6–25.7] | 0.011 |
| ASA Physical Status | 2 [2–2] | 2 [2–2] | 0.013 |
| **Comorbidities** | | | |
| Maintenance dialysis, n (%) | 271 (1.1) | 98 (0.9) | 0.022 |
| Congestive heart failure, n (%) | 1,167 (4.7) | 438 (3.9) | 0.039 |
| Malignancy, n (%) | 9,115 (37.0) | 4,210 (38.0) | 0.019 |
| **Preoperative laboratory values** | | | |
| Serum albumin, g/dL | 4.2 [3.9–4.4] | 4.2 [3.9–4.5] | 0.033 |
| Blood urea nitrogen, mg/dL | 14.4 [11.7–18.0] | 14.4 [11.7–18.0] | 0.020 |
| C-reactive protein, mg/dL | 0.09 [0.04–0.30] | 0.10 [0.04–0.31] | 0.028 |
| Serum creatinine, mg/dL | 0.77 [0.64–0.94] | 0.76 [0.63–0.92] | 0.029 |
| Hemoglobin, g/dL | 13.3 [12.1–14.3] | 13.2 [12.0–14.3] | 0.030 |
| Serum potassium, mEq/L | 4.2 [3.9–4.4] | 4.2 [4.0–4.5] | 0.120 |
| Serum sodium, mEq/L | 141 [140–143] | 142 [140–143] | 0.156 |
| Platelet count, ×10³/µL | 237.0 [198.0–282.0] | 236.0 [197.0–279.0] | 0.019 |
| Total bilirubin, mg/dL | 0.57 [0.42–0.76] | 0.57 [0.42–0.75] | 0.024 |
| White blood cell count, ×10³/µL | 6 [5–7] | 6 [5–7] | 0.008 |
| **Preoperative medications** | | | |
| Any diabetes medication, n (%) | 3,417 (13.9) | 1,583 (14.3) | 0.011 |
| Beta-blocker, n (%) | 2,464 (10.0) | 1,036 (9.3) | 0.023 |
| Oral corticosteroid, n (%) | 1,638 (6.7) | 632 (5.7) | 0.040 |
| Antiplatelet agent, n (%) | 1,337 (5.4) | 524 (4.7) | 0.032 |
| Anticoagulant, n (%) | 1,284 (5.2) | 532 (4.8) | 0.019 |
| Calcium-channel blocker, n (%) | 1,812 (7.4) | 798 (7.2) | 0.007 |
| Opioid, n (%) | 496 (2.0) | 211 (1.9) | 0.008 |
| **Procedure characteristics** | | | |
| Ophthalmic procedure, n (%) | 444 (1.8) | 113 (1.0) | 0.067 |
| Face or neck procedure, n (%) | 1,468 (6.0) | 638 (5.8) | 0.009 |
| Thoracic procedure, n (%) | 3,137 (12.7) | 1,453 (13.1) | 0.010 |
| Musculoskeletal procedure, n (%) | 4,069 (16.5) | 1,750 (15.8) | 0.021 |
| Otorhinolaryngologic procedure, n (%) | 1,397 (5.7) | 632 (5.7) | 0.001 |
| Neurosurgical procedure, n (%) | 964 (3.9) | 419 (3.8) | 0.007 |
| Genital procedure, n (%) | 3,243 (13.2) | 1,464 (13.2) | 0.001 |
| Urinary tract procedure, n (%) | 2,195 (8.9) | 1,036 (9.3) | 0.015 |
| Skin or soft-tissue procedure, n (%) | 1,790 (7.3) | 875 (7.9) | 0.023 |
| Abdominal procedure, n (%) | 6,424 (26.1) | 3,016 (27.2) | 0.025 |
| Number of resections | 1 [1–1] | 1 [1–1] | 0.039 |
| High-risk procedure, n (%) | 1,426 (5.8) | 620 (5.6) | 0.009 |
| **Intraoperative variables** | | | |
| Operative duration, min | 132 [83–204] | 131 [84–212] | 0.033 |
| Red blood cell transfusion, units | 0 [0–0] | 0 [0–0] | 0.044 |
| Fresh frozen plasma transfusion, units | 0 [0–0] | 0 [0–0] | 0.028 |
| Platelet transfusion, units | 0 [0–0] | 0 [0–0] | 0.020 |
| Intraoperative fluid balance, mL | 965 [640–1,566] | 900 [600–1,484] | 0.086 |
| **Early postoperative physiological variables** | | | |
| Heart rate at 6 hours, beats/min | 76 [67–85] | 75 [67–84] | 0.060 |
| Mean arterial pressure at 6 hours, mm Hg | 88 [79–97] | 89 [80–99] | 0.085 |

**Notes:** Data are presented as median [IQR] or n (%). Statistics were calculated from observed data before imputation. SMDs are absolute standardized mean differences between the temporal development and validation cohorts.

**Abbreviations:** ASA, American Society of Anesthesiologists; IQR, interquartile range; SMD, standardized mean difference.

**Table S6. Facility-specific performance of preoperative and perioperative prediction models in internal-external cross-validation**

| **Institution** | **Feature set** | **Model** | **N** | **Events, n (%)** | **AUROC (95% CI)** | **PR-AUC (95% CI)** | **Brier score (95% CI)** | **Scaled Brier score (95% CI)** | **Calibration slope (95% CI)** | **Calibration intercept (95% CI)** |
| --- | --- | --- | --- | --- | --- | --- | --- | --- | --- | --- |
| A | Preoperative | Logistic regression | 20,304 | 2,191 (10.8) | 0.786 (0.774–0.797) | 0.344 (0.327–0.363) | 0.082 (0.081–0.084) | 0.145 (0.131–0.158) | 0.923 (0.872–0.975) | −0.169 (−0.264 to −0.078) |
| A | Perioperative | Logistic regression | 20,304 | 2,191 (10.8) | 0.815 (0.805–0.825) | 0.410 (0.391–0.431) | 0.078 (0.076–0.080) | 0.190 (0.173–0.207) | 0.911 (0.869–0.953) | −0.353 (−0.419 to −0.285) |
| A | Preoperative | XGBoost | 20,304 | 2,191 (10.8) | 0.785 (0.774–0.795) | 0.339 (0.323–0.357) | 0.083 (0.081–0.084) | 0.140 (0.126–0.154) | 0.886 (0.839–0.931) | −0.241 (−0.324 to −0.162) |
| A | Perioperative | XGBoost | 20,304 | 2,191 (10.8) | 0.815 (0.805–0.825) | 0.414 (0.394–0.435) | 0.078 (0.077–0.080) | 0.187 (0.170–0.204) | 0.873 (0.824–0.924) | −0.408 (−0.487 to −0.330) |
| A | Preoperative | LightGBM | 20,304 | 2,191 (10.8) | 0.781 (0.770–0.792) | 0.332 (0.314–0.350) | 0.084 (0.082–0.085) | 0.131 (0.116–0.146) | 0.925 (0.877–0.978) | −0.309 (−0.388 to −0.225) |
| A | Perioperative | LightGBM | 20,304 | 2,191 (10.8) | 0.813 (0.804–0.823) | 0.400 (0.380–0.420) | 0.080 (0.078–0.082) | 0.168 (0.151–0.185) | 0.903 (0.856–0.949) | −0.480 (−0.548 to −0.412) |
| B | Preoperative | Logistic regression | 9,454 | 1,115 (11.8) | 0.760 (0.744–0.775) | 0.356 (0.332–0.381) | 0.090 (0.088–0.092) | 0.137 (0.117–0.156) | 0.824 (0.770–0.874) | −0.210 (−0.308 to −0.121) |
| B | Perioperative | Logistic regression | 9,454 | 1,115 (11.8) | 0.785 (0.771–0.801) | 0.406 (0.380–0.436) | 0.086 (0.084–0.089) | 0.171 (0.148–0.194) | 0.788 (0.743–0.841) | −0.176 (−0.266 to −0.075) |
| B | Preoperative | XGBoost | 9,454 | 1,115 (11.8) | 0.773 (0.759–0.788) | 0.373 (0.348–0.400) | 0.089 (0.087–0.091) | 0.145 (0.125–0.166) | 0.848 (0.798–0.904) | −0.218 (−0.304 to −0.120) |
| B | Perioperative | XGBoost | 9,454 | 1,115 (11.8) | 0.800 (0.786–0.813) | 0.421 (0.395–0.446) | 0.085 (0.083–0.088) | 0.182 (0.159–0.204) | 0.834 (0.786–0.881) | −0.163 (−0.245 to −0.083) |
| B | Preoperative | LightGBM | 9,454 | 1,115 (11.8) | 0.772 (0.757–0.788) | 0.373 (0.347–0.400) | 0.089 (0.087–0.091) | 0.146 (0.125–0.167) | 0.864 (0.812–0.924) | −0.249 (−0.336 to −0.153) |
| B | Perioperative | LightGBM | 9,454 | 1,115 (11.8) | 0.795 (0.780–0.808) | 0.423 (0.396–0.449) | 0.085 (0.083–0.088) | 0.180 (0.157–0.202) | 0.868 (0.817–0.916) | −0.218 (−0.302 to −0.138) |
| C | Preoperative | Logistic regression | 5,944 | 578 (9.7) | 0.833 (0.815–0.850) | 0.410 (0.375–0.447) | 0.070 (0.068–0.073) | 0.200 (0.172–0.226) | 1.116 (1.038–1.201) | 0.102 (−0.033 to 0.236) |
| C | Perioperative | Logistic regression | 5,944 | 578 (9.7) | 0.862 (0.846–0.877) | 0.458 (0.423–0.498) | 0.067 (0.065–0.070) | 0.232 (0.207–0.258) | 1.238 (1.152–1.330) | 0.582 (0.423–0.760) |
| C | Preoperative | XGBoost | 5,944 | 578 (9.7) | 0.844 (0.829–0.861) | 0.382 (0.351–0.419) | 0.072 (0.069–0.075) | 0.181 (0.151–0.212) | 0.950 (0.886–1.021) | −0.266 (−0.379 to −0.151) |
| C | Perioperative | XGBoost | 5,944 | 578 (9.7) | 0.865 (0.849–0.879) | 0.391 (0.361–0.428) | 0.071 (0.068–0.074) | 0.193 (0.162–0.223) | 1.000 (0.925–1.077) | −0.062 (−0.191 to 0.087) |
| C | Preoperative | LightGBM | 5,944 | 578 (9.7) | 0.845 (0.829–0.861) | 0.390 (0.354–0.429) | 0.072 (0.069–0.074) | 0.185 (0.154–0.215) | 0.968 (0.900–1.043) | −0.273 (−0.384 to −0.151) |
| C | Perioperative | LightGBM | 5,944 | 578 (9.7) | 0.860 (0.844–0.874) | 0.394 (0.361–0.430) | 0.071 (0.069–0.074) | 0.187 (0.156–0.217) | 1.010 (0.943–1.088) | −0.101 (−0.231 to 0.038) |

**Notes:** Each row represents performance in the held-out institution during leave-one-institution-out internal-external cross-validation. Models were developed and calibrated using the other 2 institutions and applied to the held-out institution without model updating.

PR-AUC was calculated using average precision.

The 95% CIs were calculated using 1000 stratified bootstrap resamples. Lower Brier scores and higher scaled Brier scores indicate better overall probabilistic performance. A scaled Brier score of 0 indicates performance equivalent to a prevalence-based null model. The ideal calibration slope and calibration intercept are 1 and 0, respectively.

**Abbreviations:** AUROC, area under the receiver operating characteristic curve; CI, confidence interval; LightGBM, Light Gradient Boosting Machine; PR-AUC, area under the precision-recall curve; XGBoost, Extreme Gradient Boosting.

**Table S7. Performance of preoperative and perioperative prediction models in the random hold-out validation**

| **Model** | **Feature set** | **AUROC (95% CI)** | **PR-AUC (95% CI)** | **Brier score (95% CI)** | **Scaled Brier score (95% CI)** | **Calibration slope (95% CI)** | **Calibration intercept (95% CI)** |
| --- | --- | --- | --- | --- | --- | --- | --- |
| Logistic regression | Preoperative | 0.787 (0.768–0.805) | 0.363 (0.334–0.396) | 0.082 (0.079–0.084) | 0.157 (0.134–0.181) | 0.920 (0.846–0.995) | −0.137 (−0.269 to −0.006) |
| Logistic regression | Perioperative | 0.808 (0.792–0.825) | 0.410 (0.379–0.445) | 0.078 (0.076–0.081) | 0.191 (0.164–0.220) | 0.887 (0.823–0.954) | −0.182 (−0.298 to −0.071) |
| XGBoost | Preoperative | 0.802 (0.786–0.818) | 0.395 (0.363–0.430) | 0.080 (0.077–0.082) | 0.178 (0.153–0.204) | 0.943 (0.878–1.014) | −0.119 (−0.232 to −0.000) |
| XGBoost | Perioperative | 0.824 (0.808–0.841) | 0.450 (0.418–0.484) | 0.076 (0.073–0.078) | 0.221 (0.193–0.250) | 0.937 (0.876–1.004) | −0.140 (−0.243 to −0.028) |
| LightGBM | Preoperative | 0.804 (0.787–0.821) | 0.384 (0.356–0.417) | 0.080 (0.078–0.082) | 0.174 (0.150–0.198) | 0.956 (0.894–1.028) | −0.122 (−0.230 to −0.003) |
| LightGBM | Perioperative | 0.825 (0.808–0.840) | 0.449 (0.415–0.482) | 0.076 (0.073–0.079) | 0.216 (0.187–0.245) | 0.954 (0.888–1.021) | −0.180 (−0.288 to −0.069) |

**Notes:** The full cohort was stratified by composite endpoint status and randomly divided into an 80% development set and a 20% independent hold-out test set. All preprocessing, hyperparameter optimization, model fitting, and probability calibration were restricted to the development set.

PR-AUC was calculated using average precision.

The 95% CIs were calculated using 1000 stratified bootstrap resamples. Lower Brier scores and higher scaled Brier scores indicate better overall probabilistic performance. A scaled Brier score of 0 indicates performance equivalent to a prevalence-based null model. The ideal calibration slope and calibration intercept are 1 and 0, respectively.

**Abbreviations:** AUROC, area under the receiver operating characteristic curve; CI, confidence interval; LightGBM, Light Gradient Boosting Machine; PR-AUC, area under the precision-recall curve; XGBoost, Extreme Gradient Boosting.

**Table S8. Pooled internal-external cross-validation performance of LightGBM models for postoperative infection and the composite endpoint excluding postoperative infection**

| **Outcome** | **Feature set** | **Events, n (%)** | **AUROC (95% CI)** | **PR-AUC (95% CI)** | **Brier score (95% CI)** | **Scaled Brier score (95% CI)** | **Calibration slope (95% CI)** | **Calibration intercept (95% CI)** |
| --- | --- | --- | --- | --- | --- | --- | --- | --- |
| Composite endpoint excluding postoperative infection | Preoperative | 1,061 (3.0) | 0.781 (0.765–0.796) | 0.173 (0.155–0.196) | 0.0276 (0.0270–0.0282) | 0.043 (0.021–0.064) | 0.760 (0.705–0.824) | −1.016 (−1.169 to −0.847) |
| Composite endpoint excluding postoperative infection | Perioperative | 1,061 (3.0) | 0.839 (0.825–0.854) | 0.249 (0.227–0.275) | 0.0261 (0.0253–0.0270) | 0.094 (0.064–0.121) | 0.595 (0.511–0.668) | −1.436 (−1.707 to −1.210) |
| Postoperative infection | Preoperative | 3,473 (9.7) | 0.788 (0.781–0.797) | 0.320 (0.308–0.335) | 0.0765 (0.0756–0.0775) | 0.128 (0.118–0.140) | 0.905 (0.872–0.940) | −0.336 (−0.393 to −0.280) |
| Postoperative infection | Perioperative | 3,473 (9.7) | 0.809 (0.801–0.816) | 0.344 (0.332–0.360) | 0.0752 (0.0742–0.0762) | 0.144 (0.133–0.156) | 0.902 (0.869–0.938) | −0.398 (−0.447 to −0.347) |

**Notes:** The pooled analysis included 35,702 surgical admissions and combined out-of-sample predictions from the 3 held-out institutions. The composite endpoint excluding postoperative infection comprised in-hospital death, unplanned reoperation, new renal replacement therapy, and prolonged postoperative mechanical ventilation. Patients with postoperative infection remained eligible and were classified as having the outcome if they experienced at least 1 of these components.

The preoperative model included only predictors available before surgery. The perioperative model additionally included intraoperative predictors and early postoperative predictors available within 6 hours after surgery.

PR-AUC was calculated using average precision.

The 95% CIs were calculated using 3000 stratified bootstrap resamples. A scaled Brier score of 0 indicates performance equivalent to a prevalence-based null model, whereas a value of 1 indicates perfect prediction. The ideal calibration slope and calibration intercept are 1 and 0, respectively.

**Abbreviations:** AUROC, area under the receiver operating characteristic curve; CI, confidence interval; LightGBM, Light Gradient Boosting Machine; PR-AUC, area under the precision-recall curve.

**Table S9. Multiple-imputation sensitivity analysis of LightGBM models using pooled out-of-sample predictions from internal-external cross-validation**

| **Feature set** | **AUROC (95% CI)** | **PR-AUC (95% CI)** | **Brier score (95% CI)** | **Scaled Brier score (95% CI)** | **Calibration slope (95% CI)** | **Calibration intercept (95% CI)** |
| --- | --- | --- | --- | --- | --- | --- |
| Preoperative | 0.788 (0.781–0.796) | 0.364 (0.350–0.379) | 0.083 (0.082–0.084) | 0.145 (0.134–0.156) | 0.919 (0.886–0.951) | −0.275 (−0.328 to −0.218) |
| Perioperative | 0.814 (0.807–0.821) | 0.413 (0.397–0.429) | 0.080 (0.078–0.081) | 0.178 (0.164–0.191) | 0.917 (0.887–0.947) | −0.344 (−0.388 to −0.300) |

**Notes:** Ten stochastic imputations were generated using the scikit-learn IterativeImputer with BayesianRidge as the conditional estimator, posterior sampling enabled, a maximum of 5 iterations, and 20 nearest features. Within each internal-external cross-validation fold, imputation and all data-dependent preprocessing procedures were fitted using the development facilities only and applied to the held-out facility without re-estimation. The LightGBM hyperparameters selected in the corresponding primary internal-external cross-validation analysis were held fixed. Model fitting and 3-fold out-of-fold isotonic calibration were performed separately within each imputed data set. Patient-level calibrated probabilities were averaged across the 10 imputations before performance was evaluated using pooled held-out predictions. The 95% CIs were calculated using 1000 stratified bootstrap resamples of the averaged predictions.

PR-AUC was calculated using average precision. Lower Brier scores and higher scaled Brier scores indicate better overall probabilistic performance. A scaled Brier score of 0 indicates performance equivalent to a prevalence-based null model. The ideal calibration slope and calibration intercept are 1 and 0, respectively.

Abbreviations: AUROC, area under the receiver operating characteristic curve; CI, confidence interval; LightGBM, Light Gradient Boosting Machine; PR-AUC, area under the precision-recall curve.

**Table S10. Temporal validation performance of preoperative and perioperative prediction models**

| **Model** | **Feature set** | **AUROC (95% CI)** | **PR-AUC (95% CI)** | **Brier score (95% CI)** | **Scaled Brier score (95% CI)** | **Calibration slope (95% CI)** | **Calibration intercept (95% CI)** |
| --- | --- | --- | --- | --- | --- | --- | --- |
| Logistic regression | Preoperative | 0.802 (0.788–0.815) | 0.374 (0.352–0.399) | 0.080 (0.078–0.081) | 0.168 (0.152–0.187) | 1.012 (0.956–1.071) | 0.013 (−0.084 to 0.118) |
| Logistic regression | Perioperative | 0.826 (0.812–0.838) | 0.424 (0.401–0.453) | 0.076 (0.074–0.078) | 0.206 (0.186–0.226) | 1.000 (0.945–1.058) | 0.034 (−0.064 to 0.132) |
| XGBoost | Preoperative | 0.810 (0.794–0.823) | 0.399 (0.373–0.425) | 0.078 (0.076–0.080) | 0.183 (0.164–0.202) | 1.010 (0.948–1.069) | −0.009 (−0.110 to 0.096) |
| XGBoost | Perioperative | 0.836 (0.823–0.847) | 0.450 (0.427–0.479) | 0.074 (0.072–0.076) | 0.226 (0.207–0.247) | 1.026 (0.971–1.086) | −0.027 (−0.119 to 0.074) |
| LightGBM | Preoperative | 0.810 (0.796–0.823) | 0.394 (0.368–0.420) | 0.078 (0.077–0.080) | 0.182 (0.161–0.201) | 1.031 (0.971–1.092) | −0.052 (−0.149 to 0.050) |
| LightGBM | Perioperative | 0.838 (0.826–0.849) | 0.466 (0.439–0.492) | 0.073 (0.071–0.075) | 0.234 (0.213–0.255) | 1.048 (0.990–1.107) | −0.045 (−0.137 to 0.055) |

**Notes:** Models were developed using data from January 2021 through December 2023 and evaluated without model updating in patients treated from January 2024 through March 2025. All preprocessing, hyperparameter optimization, model fitting, and probability calibration were restricted to the temporal development cohort.

PR-AUC was calculated using average precision.

The 95% CIs were calculated using 1000 stratified bootstrap resamples. Lower Brier scores and higher scaled Brier scores indicate better overall probabilistic performance. A scaled Brier score of 0 indicates performance equivalent to a prevalence-based null model. The ideal calibration slope and calibration intercept are 1 and 0, respectively.

**Abbreviations:** AUROC, area under the receiver operating characteristic curve; CI, confidence interval; LightGBM, Light Gradient Boosting Machine; PR-AUC, area under the precision-recall curve; XGBoost, Extreme Gradient Boosting.
