# Supplementary material for: Machine Learning–Based Prediction of Major Postoperative Complications After Noncardiac Surgery Under General Anesthesia Using Routinely Available Electronic Health Record Data: Retrospective Multicenter Development and Validation Study
Source: JMIR Perioper Med. 2026 Oct 21;9:e91315. doi: 10.2196/91315 (PMC13724130; doi:10.2196/91315)
Supplement: Supplementary file 2 [file periop-v9-e91315-s003.docx]

**Figure S1: Distribution of Transfused Units by Component.**

This box plot shows the median (central line), interquartile range (IQR, the box), and range (whiskers) of units transfused for Fresh Frozen Plasma (FFP), Platelets, and Red Blood Cells (RBC). The median and IQR are also labeled numerically for each component.
**Figure S2. Calibration plots of preoperative and perioperative prediction models in leave-one-facility-out internal-external cross-validation.**

Observed event rates are plotted against mean predicted probabilities within deciles of predicted risk for (A) held-out Facility A, (B) held-out Facility B, (C) held-out Facility C, and (D) pooled out-of-sample predictions from all 3 internal-external cross-validation rounds. Blue, orange, and green lines represent logistic regression, XGBoost, and LightGBM, respectively. Dotted lines with open circles represent models using the preoperative feature set, whereas solid lines with filled squares represent models using the perioperative feature set. The gray dashed diagonal indicates perfect calibration. The sample size and number and proportion of events are shown above each panel. In each validation round, models were developed and calibrated using data from the other 2 facilities and applied to the held-out facility without model updating. The pooled panel combines the held-out predictions from all 3 validation rounds.

**Abbreviations:** IECV, internal-external cross-validation; LightGBM, Light Gradient Boosting Machine; XGBoost, Extreme Gradient Boosting.

**Figure S3. SHAP summary plots for the preoperative and perioperative logistic regression models.**

Global SHAP summary plots are shown for (A) the preoperative logistic regression model and (B) the perioperative logistic regression model. Features are ranked in descending order of importance according to their mean absolute SHAP values. Each point represents 1 sampled patient from the pooled held-out observations generated across the 3 internal-external cross-validation rounds. Up to 2000 patients were sampled from each held-out facility using stratified random sampling by outcome status. The horizontal position represents the SHAP value on the log-odds scale; positive values shift the model prediction toward a higher risk of the primary composite endpoint, whereas negative values shift the prediction toward a lower risk. Point color represents the observed feature value, ranging from low in blue to high in red. SHAP values were calculated for the uncalibrated base-model output because probability calibration was applied as a separate postmodel procedure. SHAP values represent model-based predictive contributions and should not be interpreted as causal effects.

Abbreviations: ASA-PS, American Society of Anesthesiologists Physical Status; SHAP, SHapley Additive exPlanations.

**Figure S4. SHAP summary plots for the preoperative and perioperative XGBoost models.**

Global SHAP summary plots are shown for (A) the preoperative XGBoost model and (B) the perioperative XGBoost model. Features are ranked in descending order of importance according to their mean absolute SHAP values. Each point represents 1 sampled patient from the pooled held-out observations generated across the 3 internal-external cross-validation rounds. Up to 2000 patients were sampled from each held-out facility using stratified random sampling by outcome status. The horizontal position represents the SHAP value on the raw margin scale; positive values shift the model prediction toward a higher risk of the primary composite endpoint, whereas negative values shift the prediction toward a lower risk. Point color represents the observed feature value, ranging from low in blue to high in red. SHAP values were calculated for the uncalibrated base-model output because probability calibration was applied as a separate postmodel procedure. SHAP values represent model-based predictive contributions and should not be interpreted as causal effects.

Abbreviations: ASA-PS, American Society of Anesthesiologists Physical Status; SHAP, SHapley Additive exPlanations; XGBoost, Extreme Gradient Boosting.

**Figure S5. Pairwise SHAP interaction heatmaps for the preoperative and perioperative tree-based models.**

Pairwise SHAP interaction heatmaps are shown for (A) the preoperative XGBoost model, (B) the preoperative LightGBM model, (C) the perioperative XGBoost model, and (D) the perioperative LightGBM model. Each cell represents the mean absolute pairwise SHAP interaction value across sampled pooled held-out observations from the 3 internal-external cross-validation rounds. Higher values indicate stronger model-based interactions between the corresponding predictor pair. Diagonal elements representing main effects were set to 0 and are shown in white. For visualization, interaction values below the within-model 80th percentile were masked, and the 15 predictors with the greatest total interaction strength were displayed. Color scales differ among panels; therefore, interaction magnitudes should not be compared directly across models. SHAP interaction values represent predictive interaction patterns within the fitted models and should not be interpreted as causal or biological interactions.

**Abbreviations:** Alb, serum albumin; ASA-PS, American Society of Anesthesiologists Physical Status; BMI, body mass index; BUN, blood urea nitrogen; Cre, serum creatinine; CRP, C-reactive protein; Hb, hemoglobin; Malig, malignancy; PLT, platelet transfusion volume; Proc-Abd, abdominal procedure; Proc-MSK, musculoskeletal procedure; Proc-Neuro, neurosurgical procedure; Proc-Thorax, thoracic procedure; SHAP, SHapley Additive exPlanations; T-Bil, total bilirubin; WBC, white blood cell count; XGBoost, Extreme Gradient Boosting; LightGBM, Light Gradient Boosting Machine.
